# Review and Prospects on the Ecotoxicity of Mixtures of Nanoparticles and Hybrid Nanomaterials

**DOI:** 10.1021/acs.est.2c03333

**Published:** 2022-10-05

**Authors:** Fan Zhang, Zhuang Wang, Willie J. G. M. Peijnenburg, Martina G. Vijver

**Affiliations:** † Institute of Environmental Sciences (CML), Leiden University, Leiden2300 RA, The Netherlands; ‡ Collaborative Innovation Center of Atmospheric Environment and Equipment Technology, Jiangsu Key Laboratory of Atmospheric Environment Monitoring and Pollution Control, School of Environmental Science and Engineering, Nanjing University of Information Science and Technology, Nanjing210044, People’s Republic of China; § Centre for Safety of Substances and Products, National Institute of Public Health and the Environment (RIVM), Bilthoven3720 BA, The Netherlands

**Keywords:** Nanosafety, Mixture toxicity, Nanotechnology, Multicomponent nanomaterials, Independent joint action

## Abstract

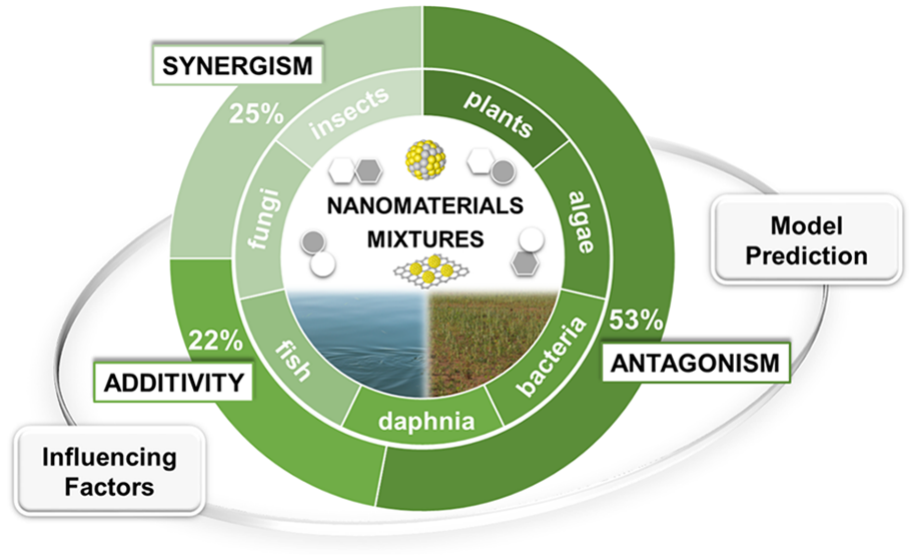

The rapid development of nanomaterials (NMs) and the emergence of new multicomponent NMs will inevitably lead to simultaneous exposure of organisms to multiple engineered nanoparticles (ENPs) at varying exposure levels. Understanding the joint impacts of multiple ENPs and predicting the toxicity of mixtures of ENPs are therefore evidently of importance. We reviewed the toxicity of mixtures of ENPs to a variety of different species, covering algae, bacteria, daphnia, fish, fungi, insects, and plants. Most studies used the independent-action (IA)-based model to assess the type of joint effects. Using co-occurrence networks, it was revealed that 53% of the cases with specific joint response showed antagonistic, 25% synergistic, and 22% additive effects. The combination of nCuO and nZnO exhibited the strongest interactions in each type of joint interaction. Compared with other species, plants exposed to multiple ENPs were more likely to experience antagonistic effects. The main factors influencing the joint response type of the mixtures were (1) the chemical composition of individual components in mixtures, (2) the stability of suspensions of mixed ENPs, (3) the type and trophic level of the individual organisms tested, (4) the biological level of organization (population, communities, ecosystems), (5) the exposure concentrations and time, (6) the endpoint of toxicity, and (7) the abiotic field conditions (e.g., pH, ionic strength, natural organic matter). This knowledge is critical in developing efficient strategies for the assessment of the hazards induced by combined exposure to multiple ENPs in complex environments. In addition, this knowledge of the joint effects of multiple ENPs assists in the effective prediction of hybrid NMs.

## 1 Introduction

Nanotechnology has undergone enormous developments recently.^1−4^ With the uninterrupted development of new emerging nanomaterials (NMs), engineered nanoparticles (ENPs) are becoming potential environmental pollutants.^5^ Mixtures of ENPs can occur due to multiple single-component NMs entering an ecosystem.^6^ Mixtures of individual ENPs have been detected within municipal wastewater treatment systems^7−11^ and subsequently in the receiving waters and soils. Mixtures of individual ENPs may harm aquatic and terrestrial species (including humans) by coaccumulating in the food chain. Considering that multiple distinct ENPs may coexist in the same environmental compartments, it is critical to determine how mixtures of individual ENPs may affect environmental receptors. Additionally, multicomponent NMs, so-called hybrid or advanced NMs, are by definition a mixture but need to be distinguished from mixtures of individual ENPs. There currently is a clear trend of technological innovations moving toward the development of more complex advanced materials. However, limited information is available on the occurrence, fate, and toxicity of mixtures of NMs as well as for multicomponent NMs in the environment. It thus is imperative to perform studies that characterize the hazards of hybrid NMs at an early stage of their development, starting at the research phase. The knowledge built from mixtures of NMs can be used to get an estimate of the (magnitude of) quantification of the joint impacts of multiple elements and particles. There is also an urgent need for extrapolating knowledge gained on individual ENPs toward hybrid NMs. This will minimize undesirable impacts on human and environmental health at later stages of development and production and will allow a conscious move toward sustainable nanotechnology and responsible innovation.^12^

Assessing the joint impacts of chemicals is already notoriously difficult, and for ENPs this could be even more challenging. After all, the chemical composition and the particle characteristics need to be accounted for. Subsequently, the toxicity of NMs is inherently composed of the toxicity of the particle constituents as well as the particle-specific fate and toxicity. Analyzing the scattered experimental data on mixtures of ENPs will lead to a better understanding and will allow verification of whether conventional mixture models can be used to describe joint impacts of NMs.^13^

In this paper, we therefore addressed the following subresearch questions. (1) What joint interactions have been reported after exposure of a range of aquatic and terrestrial test species to multiple ENPs? (2) Which factors determine the toxicity of a mixture of multiple ENPs? (3) Is there a difference between the environmental behavior and fate of multiple ENPs compared to single ENPs and do such differences subsequently affect the induced ecotoxicological effects? (4) Which important knowledge gaps and further research needs have been identified in assessing mixture-nanoecotoxicology for experimentalists, computational modelers, risk assessors, and regulators? To address these scientific questions, we have collated information on the mixture toxicity of ENPs spanning trophic levels as well as aquatic and terrestrial environments available in the literature. Herein, we focus on two types of multiple ENPs, namely mixtures of individual ENPs and hybrid NMs. Meanwhile, the nanohybrids of concern are mainly synthetic materials with organic or inorganic ENP components that are linked together by noncovalent bonds or covalent bonds at the nanometer scale. The strength of the joint interactions of multiple ENPs and the main factors influencing the joint response of the mixtures were identified for the first time in this work. Ultimately this knowledge constitutes the first building blocks that allow building a computational approach able to reduce the experimental costs of ecotoxicity testing of mixtures of ENPs of varying composition and to include both nanohybrids and mixtures of different ENPs.

## 2 Methods

Data were mined from peer-reviewed articles as published between 2003 and 2022, making use of the search machines Web of Science and PubMed (last access date March 10th, 2022). The inclusion criteria were as follows: (Toxicity OR Ecotoxicity) AND (Nanomaterial* OR Nanoparticle* OR Nanoplastic*) AND (Mixture* OR hybrid) AND (Alga* OR Bacteria* OR Daphnia OR Fish OR Insect* OR Plant*).

On the basis of these search terms, we obtained 1263 publications and removed duplicate papers as well as those in which the title, abstract, or text was not related to the toxicity of mixtures of NMs to ecological species (e.g., papers on microsized plastic particles). A final total of 86 papers were filtered and extracted for future reviewing, as shown in Figure S1.

Data were collected for representative ecological species (algae, bacteria, daphnia, fish, fungi, insects, and plants). Binary and ternary ENP toxicity data reported from laboratory-derived studies were collected, as well as effect data on nanohybrids. The types of joint interactions (additive, synergistic, and antagonistic) of the mixtures of ENPs given in the original literature were extracted from the eligible papers. The mixtures induced additive effects or deviated from additivity, either by synergistic (toxicity of the mixture higher than the summed toxicity of the individual ENPs) or antagonistic (toxicity of the mixture lower than the summed toxicity of the individual ENPs) mixture toxicity.

In the selected papers, three common concepts enabling to assess mixture toxicity—concentration addition (CA), independent action (IA), and toxic unit (TU)—were used. In addition to assessing the impacts of the mixtures, the abiotic conditions expected to influence toxicity and information on the existing predictive methods for evaluating the mixture toxicity were collected as well.

Following the evaluation of the first 86 papers, an association rule analysis (which is a technique to uncover how items are associated with each other) was performed to mine the literature data. Calculated networks based on co-occurrence explain which combination of NMs has been most studied, which combination of NMs is more likely to have an additive, synergistic, or antagonistic effect, which species are more sensitive to additive, synergistic, or antagonistic effects, and which method is commonly used in assessing the joint toxicity of multi-ENP mixtures. The association rule analysis was performed using the Apriori algorithm in the classification of association rule in IBM SPSS Modeler (ver. 18.0) and was further visualized using Cytoscape (ver. 3.9.0).

## 3 Results and Discussion

### 3.1 Types of Joint Interactions of Multiple ENPs

**Figure 1:**
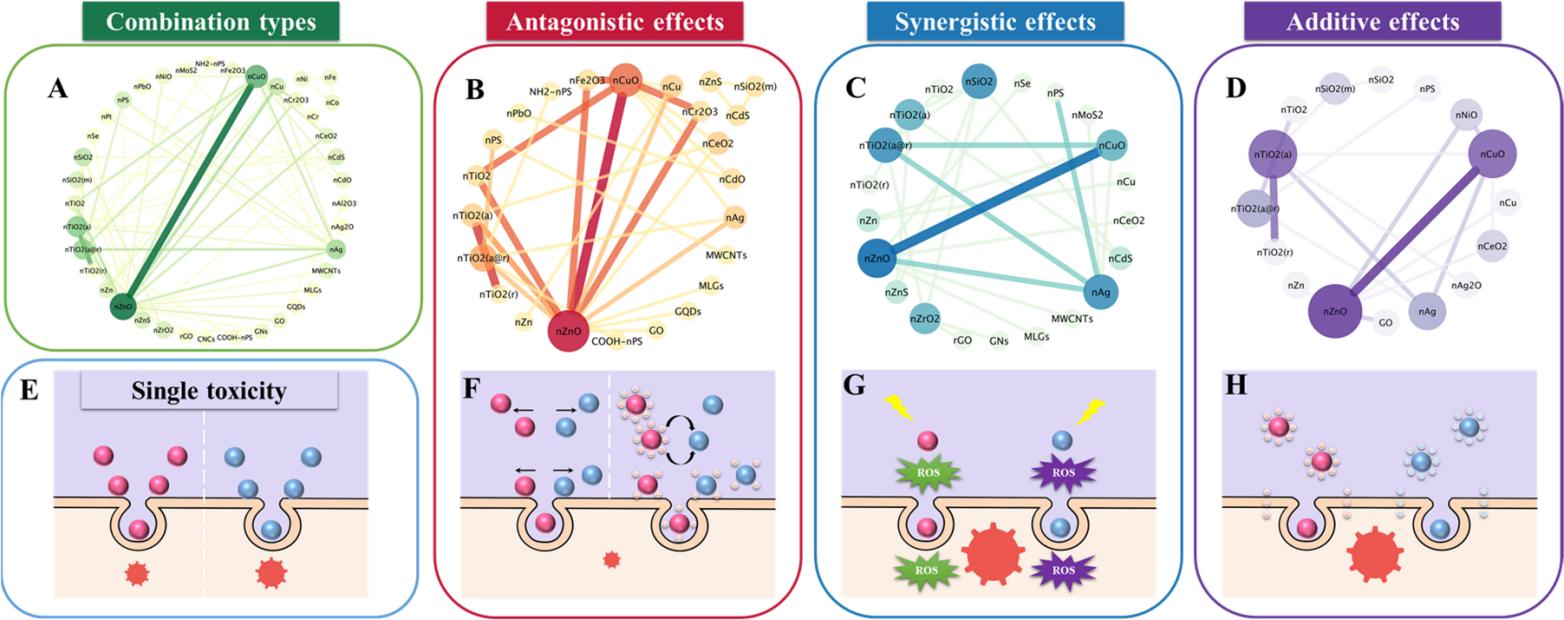
Co-occurrence network showing the correlations between different ENPs (A–D) and illustration of the main mechanisms of single toxicity (E) and joint interactions (F, antagonism; G, synergism; H, additivity) of mixtures of individual ENPs.

The data in Tables S1 and S2 illustrate the different combination types of individual ENPs, ecological species, test concentrations and mixture ratios, endpoints, and intentions in joint action analyses of mixtures. Figure 1A depicts a network that connects ENPs in different combinations on the basis of the data gathered from the literature (Tables S1 and S2). The binary mixture of nCuO and nZnO is the most studied combination in the available reports, as indicated in Figure 1A. As is known, nCuO and nZnO are among the most produced and commonly used ENPs.^14^ In addition, frequently studied combinations are nTiO_2_ (anatase) + nTiO_2_ (rutile) and nCu + nZnO in order of preference. Generally, at the current stage, studies have mainly focused on examining the toxicity of mixtures of metal-based ENPs (75% of all combinations).

Figure 1B–D depicts a network that connects ENPs in different types of joint interactions, on the basis of the data gathered from the literature (Tables S1 and S2). In all combinations with a known joint response, 53% of the interactions induced antagonistic effects, 25% of the interactions induced synergistic effects, and 22% of the interactions were additive. In addition, note that the same combinations such as nCuO and nZnO might induce antagonistic and synergistic as well as additive effects. It is important to note that the reported data involved both aquatic and terrestrial environments and different trophic levels. Following that, the prevalent concentration levels, bioavailability, and physical–chemical behavior of ENPs in mixtures and present as hybrids vary in different compartments. The effects of the mixtures could potentially be affected by this inherent difference with regard to the fate of ENPs in the environmental compartments. The interaction strengths that were found by using a co-occurrence network analysis (Figure 1B–D) are described in detail below.

#### 3.1.1 Antagonistic Effects

Antagonism is the most common mode of joint interactions of multiple ENPs observed in the current studies on mixture toxicity of ENPs. As shown in Figure 1B, nCuO showed the strongest antagonistic interactions with nZnO. The nTiO_2_ (anatase) and nTiO_2_ (rutile) combination was also found to be more inclined to show antagonistic effects, followed by nCr_2_O_3_ + nZnO, nCuO + nCr_2_O_3_, nCuO + nFe_2_O_3_, nCuO + nTiO_2_, nFe_2_O_3_ + nZnO, and nTiO_2_ + nZnO. In most instances, the occurrence of antagonistic responses implies that the presence of one ENP component in a mixture reduces the uptake of other ENP components by an organism or allows for adsorption of toxic metal ions released by the dissolution of other ENP components (Figure 1E,F). This leads in turn to an overall reduction of the toxicity of the mixture. For example, the combined toxicity of nCu and nCuO to the luminescent bacterium *Vibrio fischeri* is antagonistic, and this joint response is associated with the saturation of Cu uptake by the bioreceptor.^15^ This differs from the general assumption that an additive effect is expected as both nCu and nCuO release Cu ions. This assumption tends to take into account only the intrinsic properties of the ENPs and does not take into account the interactions between the mixed components and the interactions between organisms and ENPs. The binary mixtures of nCu and nZnO exhibit antagonistic effects on *V. fischeri*, which is associated with the adsorption of nCu ions released by dissolution of nCu onto nZnO.^15^ Yu et al.^16^ found that the mode of joint toxic action of nCeO_2_ and nTiO_2_ against *Nitrosomonas europaea* was antagonistic, and the impacts of nCeO_2_ were mitigated as a function of the exposure dose of nTiO_2_. As both negatively charged nCeO_2_ and nTiO_2_ particles can interact with bacterial cells, and as the electrostatic repulsion between the particles may prevent their coagglomeration/aggregation, the two nanoparticles may compete for adsorption sites on the cell wall, thus mitigating the toxic effect of nCeO_2_ exposed solely.^16^

#### 3.1.2 Synergistic Effects

As shown in Figure 1C, the coexistence of nCuO and nZnO also showed the strongest synergistic interactions among all of the combinations with known synergistic effects. The interactions between nAg and polystyrene nanoplastics (nPS), nAg and nTiO_2_ (anatase@rutile), nAg and nZnO, and nCuO and nTiO_2_ (anatase@rutile) are slightly weaker than the interaction between nCuO and nZnO. The synergistic effects of ENPs can be largely due to the fact that they synergistically induce elevated levels of reactive oxygen species (ROS) (Figures 1E,G). For example, the synergistic effect of exposure of *Escherichia coli* to a mixture of nAg and nTiO_2_ was associated with enhanced photocatalytic activity and elevated intracellular ROS levels.^17^ Zhang et al.^15^ also found that the effects of the binary mixtures of nCu and nZn, nCuO and nZn, and nCuO and nZnO were synergistic to *V. fischeri*. This is related to the enhancement of intracellular ROS levels induced by these mixtures. Additionally, Wang et al.^18^ addressed that the synergistic cytotoxicity induced by graphene nanoplatelets (GNs) or reduced graphene oxide (rGO) and metal-based nZrO_2_ to *Chlorella pyrenoidosa* and the mechanism underlying this synergistic action were associated with the induction of intracellular oxidative stress and cellular membrane functional changes by the carbon–metal-based mixtures. In addition, the effects of mixtures of nAg and nZnO on *Daphnia magna* were synergistic, while their respective salts (AgNO_3_ and ZnCl_2_) behaved antagonistically.^19^ This finding indicates that the dissolved ions are not always responsible for ENP toxicity but that ions + nanoparticles together can cause different effects to aquatic organisms.^19^ The synergistic effects of ENPs can be more harmful to ecologically relevant species and to human health, and there is an urgent need to examine the toxicity of mixtures of various combinations of ENPs and thus assess their potential synergistic risks.

#### 3.1.3 Additive Effects

Relatively fewer studies have reported on the combined toxicity of ENPs in an additive manner. As shown in Figure 1D, the combination of nCuO and nZnO displays stronger additive interactions than other ENP combinations. An additive effect is also frequently found in the mixtures of nTiO_2_ (anatase) and nTiO_2_ (rutile). Zhang et al.^15^ reported that a binary mixture of nZn and nZnO exhibited additive toxicity to *V. fischeri*. An analysis of the type of joint response suggested that nZn did not interact with nZnO and that the bioreceptor might not be saturated with Zn.^15^ Singh and Kumar^10^ found that a combination of nanosilver oxide (nAg_2_O) and nTiO_2_ caused additive toxicity to *Spinacia oleracea* and improved the plant biomass. In addition, graphene oxide (GO) and nZnO also exerted combined toxic effects on *D. magna* in an additive manner.^20^ The toxicity of multiple ENPs works in an additive manner in the sense that the toxicity of a mixture of individual ENPs is equal to the sum of the toxicity of each ENP component acting alone (Figure 1E,H). The additive effect is characterized by the fact that each ENP component in the mixture can proportionally substitute for another ENP component without altering the overall toxicity of the mixture. Furthermore, the additive type of joint interaction is further divided into concentration-additive and effect-additive modes. Future studies are needed to identify the types of additive modes of action in order to elucidate the main pathways by which multiple ENPs achieve additive joint interaction.

### 3.2 Potentiation or Attenuation of Effects

Some of the studies shown in Tables S1 and S2 do not directly indicate the type of joint interactions for mixtures of ENPs but imply a difference between combined and single exposures. The mixture effects caused by this scenario are expressed in detail in Table S3. Multiple ENPs cause enhanced toxic effects in a manner where one ENP in a mixture is less toxic or nontoxic to the organism, but its toxic effects are enhanced by concurrent exposure with another ENP. An example of potentiation effects was that coexposure to the binary mixtures of nCu and nZnO caused mortality of *Oncorhynchus mykiss* at no-effect concentration levels for each of the individual ENPs.^21^ The authors explained this by the higher Zn-ion accumulation in the fish when nCu was present. Collectively, the current studies indicated that the potentiation of the effects of multiple ENPs was mainly correlated with increased bioaccumulation of toxic components^22,23^ and oxidative stress.^23,24^ Conversely, an attenuated toxic effect was found by Zhao et al.,^25^ who reported that nAl_2_O_3_ was shown to mitigate the growth inhibition toxicity of GO to *C. pyrenoidosa*. Zhao et al.^25^ explained the reduced exposure of alga to GO in the presence of nAl_2_O_3_ due to GO-nAl_2_O_3_ heteroaggregation. Evidently, the proposed reason for the attenuation effect is related to coaggregation and surface complexation,^26^ a reduction in the bioavailability of toxic components,^22,27^ and oxidative stress symptoms.^22,28^ In addition, such potentiation or attenuation of effects is relative if the mixture effect lies between the effects of the individual ENPs.^29^

### 3.3 Exposure of Biota to Hybrid NMs

To date, concerns about the toxicity and safety of nanohybrids on release into the environment have also increased considerably. In particular, the strong interactions between nanoparticles in hybrid NMs (the primary concern here is that enhanced toxicity is induced when ENPs are mixed within a (crystalline) matrix of different NMs) could allow the nanocomposite to act in a mode of toxic action that may be different from the mode(s) of toxic action of a mixture that is composed of the separate nanosized components. The collected publications addressing the ecotoxicity of advanced NMs are summarized in Table S4. Generally, there is controversy about the ecotoxicity of nanohybrids. Some studies addressed that hybrid NMs show no signs of toxicity to ecological species. For instance, Da Silva et al.^30^ found that nTiO_2_ and multiwalled carbon nanotubes (MWCNTs) hybrids presented no acute toxicity to zebrafish embryos. However, most of the studies indicated that hybrid NMs exhibited diverse levels of toxic effects on ecological species.^31−33^ In particular, the minimum inhibitory concentration (MIC) of selected hybrid NMs (i.e., α-nFe_2_O_3_@nCo_3_O_4_, Chit-nAg@GO, nAg@GO, nAg@MWCNT, nAu@nAg, and rGO@nCu_2_O) to bacteria ranges from 1 to 1000 μg/mL (Table S4 and Figure S2), implying that nanohybrids could be harmful to ecological species. Moreover, hybrid NMs containing nAg and any other material with a lower MIC may provoke more toxic effects, as shown in Figure S2. Furthermore, hybrid NMs can be either more or less toxic than that where each separate component of the nanohybrid was to act on its own. This implies that the ecotoxicity of multicomponent NMs is either between^31^ or higher than the toxicities^32^ of the individual ENP components. In particular, some studies have highlighted that the enhanced bactericidal activity of binary ENP nanocomposites was the result of the synergistic effect of their individual ENP components.^34−36^ The combination of multiple NMs allows new properties to emerge and/or adds to the targeted properties.^30^ Because of this, the properties that determine the toxicity of a single NM may not be the same for multicomponent NMs. Therefore, an understanding of the risks of nanohybrids remains uncertain and needs to be clarified.

With the emergence of new hybrid NMs, such as early-transition-metal carbides and nitrides (MXene)^37^ and graphitic carbon nitride based nanohybrids,^38^ the areas of application are widening and the value of their applications is increasing.^39^ However, due to the diversity and complexity of hybrid NMs, toxicological studies and assessment methods on these hybrid materials are challenging. In particular, nanohybrids which have abundant interfaces and active sites (e.g., defects, dangling bonds, and functional groups) tend to be very sensitive and unstable in the exposure medium (being the mimicked environment). Therefore, there is an urgent need to carry out studies on the physical, chemical, and biological transformations that occur in hybrid NMs in environmental media and to determine how these transformation behaviors ultimately affect their ecotoxicity.

### 3.4 Main Factors Influencing Mixture Toxicity of Multiple ENPs

From the above results, it appears that multiple ENPs in different studies exhibit different or even opposite mixture effects. For example, the joint toxicity of nCuO and nZnO was determined to be antagonistic in most studies, while some studies determined it to be synergistic or additive. This is because the type and intensity of the joint response of multiple ENPs are influenced by a number of factors, such as chemical composition, physicochemical behavior, organismal factors, and the environmental conditions in which multiple ENPs and organisms would be located. Scientifically, the determination of the various factors influencing toxic effects is an important part of the study of mechanisms of toxic action and an important building block for exploring methods and mechanisms to reduce the biological toxicity of multiple ENPs before they are widely used or released into the environment. From an engineering perspective, it is particularly important to guide environmental remediation, which is the use of physical, chemical, and biological techniques to reduce the concentration or toxicity of pollutants present in the environment or to render them completely harmless.^40,41^ In environmental remediation, depending on the toxic factors, control can be sought to make environmental remediation efforts relevant. Therefore, there is a need to explore ways and mechanisms to reduce the toxicity of a mixture of multiple ENPs by analyzing how each factor affects the mixture toxicity.

#### 3.4.1 Chemical Composition of Mixed Components

The toxicological effects of ENPs are closely related to especially their chemical composition. Mixtures composed of ENPs of different chemical compositions also exhibit markedly different toxic effects on the same species. For example, the joint toxicity of nCuO and nCu against *V. fischeri* showed antagonistic effects, while the joint toxicity of nCuO and nZn against *V. fischeri* showed synergistic effects.^15^ Similarly, nCeO_2_ had an antagonistic toxic effect on *N. europaea* in a combination with nTiO_2_, while nCeO_2_ had a synergistic toxic action with nZnO.^16^ It can also be deduced that the presence of nTiO_2_ alleviated the toxicity of nAg to *E. coli*,^42^ whereas the presence of nPt strengthened the toxicity of nAg to *E. coli*.^43^ Moreover, the hybrid NM nAg@GO (MIC: 3.2 μg/mL^44^) is more toxic to *E. coli* than the hybrid NM nAu@nAg (MIC: 10 μg/mL^36^). The type of joint interaction between nSiO_2_ and other ENPs (nCdS, nTiO_2_, and nZnS) to *Heterosigma akashiwo* was also significantly influenced by the absence and presence of metal inclusions in nSiO_2_.^45^ In addition, the mode of joint toxic action of three metal oxide ENPs (nCuO, nCeO_2_, and nZnO) against *Carassius auratus* changes from synergistic or antagonistic to additive effects when the chemical composition of a mixture changes from a binary to a ternary mixture.^46^

#### 3.4.2 Stability of Suspensions of Mixed ENPs

The stability of suspensions of ENPs is affected by processes such as aggregation/agglomeration, dispersion, sedimentation, dissolution, and other transformations of ENPs. These processes affect the size, morphology, or form (nano or ionic) of ENPs in environmental media, and they are therefore important factors affecting the toxicity of ENPs. By means of the Derjaguine–Landaue–Verwaye–Overbeek (DLVO) theory, it was shown that the aggregation of a mixture of ENPs such as nCuO and nZnO in aquatic systems might be happening due to the combined effects of ionic layer compression, charge neutralization, and van der Waals attraction.^47^ These interaction forces drive the occurrence of coaggregation/agglomeration of multiple ENPs and also contribute to the distinct differences in their modes of joint toxic action.^16^ It has also been found that the copresence of naturally derived cellulose nanocrystals (CNCs) significantly reduced the aggregation of nZnO, resulting in enhanced bioavailability and toxicity to *Eremosphaera viridis*.^23^ Furthermore, interactions between individual ENPs in a mixture play a mediating role in ENP toxicity, particularly for a mixed system consisting of a soluble ENP such as nZnO and other stable ENPs such as nTiO_2_.^48^ The concentration of free Zn ions released from nZnO can be scavenged due to the formation of Zn(II)-TiO_2_ surface complexes, which may consequently alter the exposure and bioavailability of nZnO to organisms.^48^ This interaction would often cause antagonistic effects of multiple ENPs.^49,50^ Besides, the ability of an ENP in a mixed system to act as “Trojan horses” carrying a dissolved ion released from another soluble ENP to targeted organs and sites cannot be underestimated. This may elevate the mixture effects of individual ENPs, though the effects of such interactions on the toxicity of multiple ENPs still need further investigation.

#### 3.4.3 Types and Trophic Level of Individual Organisms Tested

**Figure 2:**
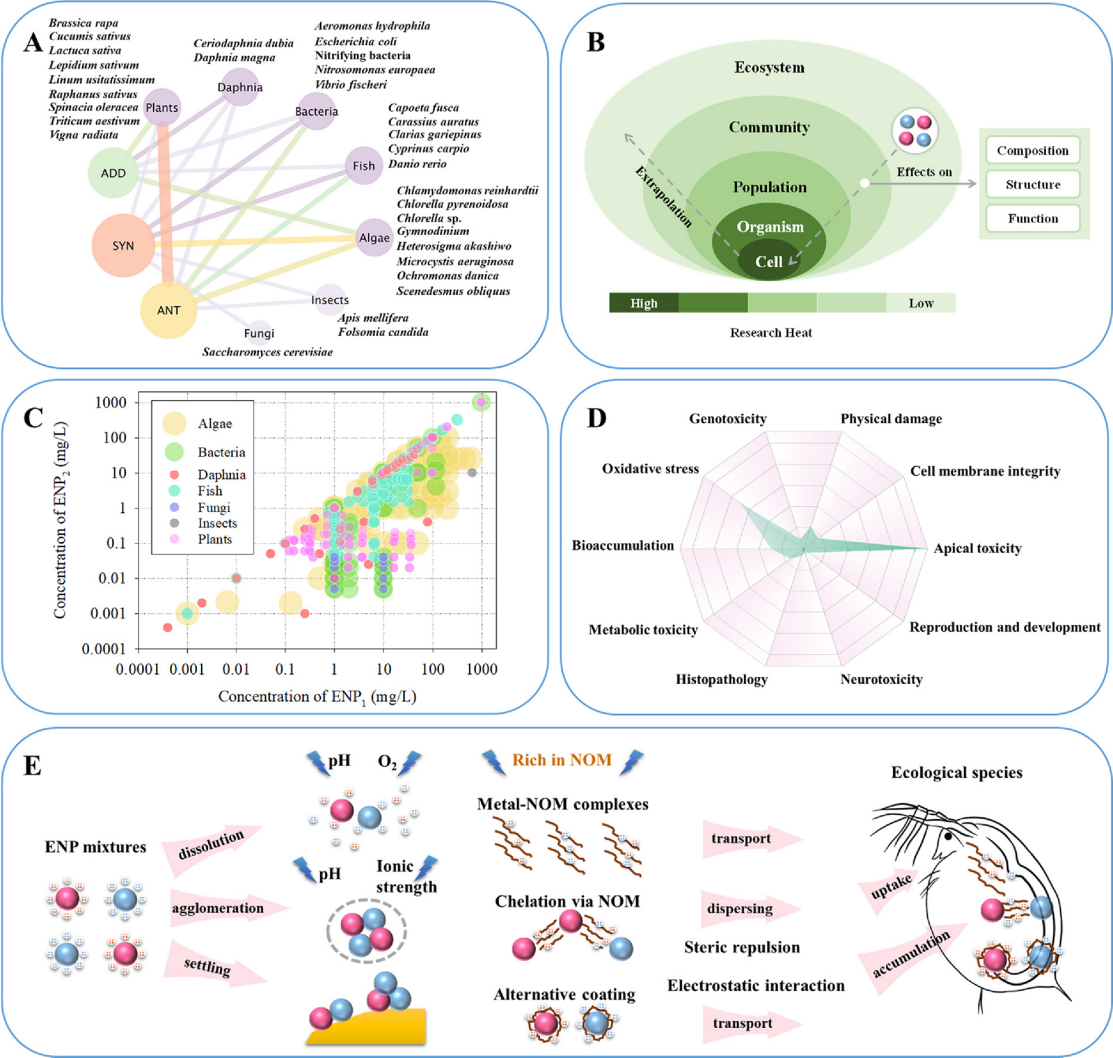
Main factors influencing mixture toxicity of multiple ENPs. (A) Network diagram of association rules of ecotoxicological test species combined with types of joint interactions of multiple ENPs (ANT, antagonism; SYN, synergism; ADD, additivity). (B) Biological levels of organization in ecosystem-relevant ecological toxicology of multiple ENPs. (C) Comparison of the ENP concentrations used in exposure studies with binary ENP mixtures. (D) Endpoints of toxicity selected in current studies on the mixture toxicity of multiple ENPs. (E) Schematic description of the effects of natural organic matter (NOM) on the toxicity of the mixture of individual ENPs.

Figure 2A depicts a network that connects tested organisms with types of joint interactions of multiple ENPs. An association analysis indicated that antagonistic effects occur particularly in plants, followed by algae. Synergistic effects frequently take place in algae. An additive effect is also mostly observed in algae and plants. For the frequency of occurrence of types of joint responses, all three types of joint interactions are observed in algae, bacteria, daphnids, fish, and plants. Furthermore, it is evaluated that 68% of the interactions are more likely to have an effect on lower trophic level organisms, including algae and plants. This means that organisms which are at lower trophic levels present more sensitivity to joint responses to the mixtures of multiple ENPs than those which are at higher trophic levels. Consequently, the trophic level may have an important impact on the mixture toxicity of multiple ENPs.

This sensitivity is particularly observed when mixtures of ENPs with the same composition exhibit different toxic effects on different species. For example, enhanced toxicity of the binary mixtures of nCu and nZnO to *Oncorhynchus mykiss* was observed,^21^ while the binary mixture showed an antagonistic effect on *V. fischeri*(15) and lettuce (*Lactuca sativa* L.).^51^ The binary mixtures of GO and nZnO had an additive toxicity against *D. magna*, while the binary mixtures had an antagonistic toxicity against zebrafish (*Danio rerio*).^20^ In addition, the joint toxicity of spherical nTiO_2_ and tubular nTiO_2_ to *C. pyrenoidosa* was observed to be significantly higher than their joint toxicity to *Scenedesmus obliquus*, and the mode of interaction of the binary mixtures of spherical nTiO_2_ and tubular nTiO_2_ to *C. pyrenoidosa* was found to be effect addition, whereas the joint toxicity to *S. obliquus* was based on concentration addition.^52^

#### 3.4.4 Biological Level of Organization

Ecotoxicological effects resulting from exposure to ENPs can be attributed to changes in the state or dynamics of biological organization, because fitness differences at individual organism levels can have a range of ecological consequences (Figure 2B). Overall, most existing nanoecotoxicological studies have focused on the cellular and individual levels, for which mortality, ROS, and reproduction rates are the most often reported endpoints for the standard laboratory species. If for at least three trophic levels (e.g., algae, daphnids, fish) data are collected, a species sensitivity distribution (SSD) curve can be generated to assess the impact of the NMs on the potential affected species at the community level. For a variety of nAg these SSDs have been calculated and reported by Chen et al.^53^ For mixtures these types of SSD curves can be calculated as well, making use of the multisubstance formulas. However, these types of SSDs have not yet been reported in the literature for mixtures of ENPs or for hybrid NMs. The main reason for this is the lack of toxicity data for sublethal effects of mixtures of NMs: i.e., the median effect concentration (EC_50_), the lowest observed effect concentration (LOEC), or data on the no observed effect concentration (NOEC) of mixtures.

Experimentally, some data have been reported on mixtures of individual ENPs, mostly how they affect microbial communities^6,11,54,55^ for a range of exposure scenarios. A river bacterial community structure was shifted significantly as a consequence of addition of nTiO_2_, nZnO, and nAg in different combinations, and with the dominant population being suppressed, the community exposed to ENPs became more diverse.^54^ Another study reported that, even at the relatively modest concentrations used, a combination of nAg, nCu, and nSiO_2_ has the potential to disrupt an arctic soil community.^55^ Additionally, a mixture of nAg_2_O and nTiO_2_ had a greater impact on activated sludge than the individual ENPs when they were present at the same concentrations.^11^ It is evident that the effect of ENP mixtures is not diminished by the increased biological level of organization. By modulating ENP properties such as ion release and shape, ENPs such as nAg can play a significant role in the functional composition of microbial communities.^56^ This warrants the consideration of the combined effects of individual ENPs with different properties on a biological community and associated ecosystem processes in environmental science and management.

#### 3.4.5 Exposure Concentrations and Time

The concentration distribution of the mixture components in the toxicity studies of the selected binary mixtures for different species is given in Figure 2C. A wide range of concentrations used for mixture toxicity testing was studied. The concentrations studied have been more focused on the range between 0.1 to 100 mg/L, which corresponds mainly to joint toxic effects on algae, bacteria, daphnia, fish, and plants. A combination of available examples found the type of joint interactions can be dependent on the doses of ENPs. For example, when the doses are close to the concentration that causes 50% of immobilization, the synergism between nAg and nZnO in *D. magna* changes to antagonism.^32^ In addition, lower mixture concentrations of nTiO_2_ (0.025 or 0.25 mg/L) and 1 mg/L nPS showed an antagonistic type of interactions in *S. obliquus*.^24^ In contrast, an additive interaction was observed between the highest concentration of nTiO_2_ (2.5 mg/L) and 1 mg/L nPS.^24^ It is evident that the ratio of exposure concentration of individual ENPs in a mixture also plays a role in determining the type of joint response.

The type of joint response for mixtures of individual ENPs is also time-dependent. For instance, the antagonistic and synergistic effects of Zn- and Cu-based ENPs on the reproduction reduction of *Folsomia candida* were observed in soil samples after 1 and 90 days, respectively.^57^ Combined treatment of ENPs triggered different physiological, chemical, and transcriptional effects on soil-grown barley *Hordeum vulgare* than those caused by individual exposure to nCuO or nZnO in a time-dependent manner.^58^ The distinct joint effects of multiple ENPs may be caused by the differences in the transformation of ENPs (e.g., aggregation/agglomeration, dissolution) over time in environmental media.

#### 3.4.6 Endpoints of Toxicity

Figure 2D depicts the endpoints of toxicity used for mixture toxicity testing. Current tests examining the toxicity of mixtures of multiple ENPs include various endpoints of toxicity, which characterize their toxic effects from the apical to the mechanistic level. In existing studies apical toxicity endpoints (e.g., growth inhibition, mortality) are used as the primary toxic endpoints for characterizing the impacts of mixtures of multiple ENPs on ecological species, as shown in Figure 2D. It can also be observed that oxidative stress has become the primary endpoint of toxicity assessment in elucidating the mechanisms of joint responses of biota to exposure to mixtures of multiple ENPs. Furthermore, the selection of toxicological endpoints has an obvious impact on the manner in which the joint responses of multiple ENPs are interpreted. For instance, multilayer graphenes (MLGs) and nZnO showed synergistic effects on *Capoeta fusca* using mortality rate as an endpoint, whereas MLGs and nZnO showed antagonistic effects on the same species when behavioral responses and histopathological changes were used as endpoints.^59^ Likewise, chitosan-functionalized molybdenum disulfide nanosheets (nMoS_2_) attenuated the oxidative stress induced by nAg on yeast cells, while nMoS_2_ had a synergistic effect with nAg in destroying the yeast cell membrane integrity.^60^ Generally, apical toxicity endpoints provide the most robust findings to describe multiple ENP toxicity.

#### 3.4.7 Field Conditions

Under different abiotic field conditions (i.e., pH, ionic strength, dissolved organic carbon, etc.), ENPs can undergo various physicochemical transformations^61^ such as dissolution, adsorption, aggregation/agglomeration, and dispersion. Each of these processes can affect the biological availability of ENPs (Figure 2E). The multi-ENP mixtures can also undergo these physicochemical transformation processes, thus affecting the fate and toxicity of individual ENPs in the mixtures.^49,51^ Understanding the extent of physicochemical transformation of multi-ENP mixtures in environmental media is therefore essential for estimating ecological risks.^62^ The extent of these transformations such as dissolution and aggregation/agglomeration will be controlled by abiotic field conditions. The aggregation and settling behavior of a mixture of ENPs such as nCuO and nZnO within aquatic systems was found to be dependent on pH, ionic strength, and concentration, and dissolution of the ENPs was observed to be significantly affected by a change in the pH of a suspension.^47^ Furthermore, the stability of suspensions containing a mixture of nCuO and nZnO was found to decrease with increasing pH, ionic strength, and ENP concentration.^47^ Another study showed that aggregation in a suspension containing a mixture of nCuO and nZnO in natural water was significantly affected by the ENP concentration, clay concentration, and humic acid.^63^

It is known that abiotic field conditions, such as UV exposure,^64^ pH,^65^ ionic strength,^66^ and natural organic matter (NOM),^65,67^ can influence how ENPs affect different organisms. Consequently, ecotoxicological testing for mixtures of ENPs should include assessment of the exposure of organisms under a variety of exposure conditions to fully represent the field conditions found in the natural environment. One critical parameter influencing chemical interactions is exposure to light. In the dark, nTiO_2_ attenuated bacterial stress caused by low concentrations of nAg due to Ag^+^ adsorption.^42^ Yet, since both nTiO_2_ and nAg are photoactive, their photochemistry may play a key role in their interactions. In a further study by Wilke et al.,^17^ the chemical interactions of nAg and nTiO_2_ mixtures in a natural aqueous medium under simulated solar irradiation were studied to investigate photoinduced stress. Wilke et al.^17^ observed that nTiO_2_ and nAg together exert synergistic toxic stress in *E. coli* by using adenosine triphosphate levels and cell membrane integrity as probes. In addition, NOM is demonstrated to be an important parameter affecting the behavior and effect of ENP mixtures. Zhao et al.^25^ found that humic acid decreased GO-Al_2_O_3_ toxicity to *C. pyrenoidosa* due to enhanced steric hindrance through a surface coating of GO-Al_2_O_3_ heteroaggregates. In contrast, Yu et al.^68^ demonstrated that Suwannee River NOM increased the relative contribution of dissolved ions released from nCu and nZnO to the toxicity of the binary mixtures at high-effect concentrations of individual ENPs to *D. magna*. Moreover, the presence of Suwannee River NOM significantly enhanced the accumulation of either nCu or nZnO in *D. magna* exposed to the ENP mixtures.^68^ As depicted in Figure 2E, the increase in the accumulation of a mixture of ENPs in the presence of NOM may be related to the direct ingestion of metal-NOM complexes and ENP-NOM complexes by water-exposed free-swimming species.

Once released into the environment, nanoparticles can also adsorb naturally occurring biomacromolecules such as secreted proteins and polysaccharides onto their surface: namely, an eco-corona formation.^69^ The presence of an eco-corona can alter the surface properties and aggregation state of nanoparticles in the aquatic environment,^70,71^ as well as alter their ecotoxicity.^72,73^ However, there is a paucity of literature reporting on the properties, patterns, and mechanisms of competitive formation of an eco-corona on multiple ENPs or formation of mixtures of individual ENP-eco-corona complexes. Consequently, the impact of eco-corona formation on the combined adverse effects of mixtures of ENPs has also become one of the scientific challenges to be solved.

Additionally, biochar as a sustainable and renewable source has been used successfully for the *in situ* remediation of various pollutants during different environmental governance processes.^74,75^ The concurrence of biochar also induces a positive effect in reducing the biotoxicity and bioavailability of ENPs.^76,77^ However, the current understanding of the interactive effects of biochar and multiple ENPs on ecological species is rather limited. The impacts of biochar on the combined toxicity of individual ENPs need to be highlighted and potential opportunities identified to maximize the understanding of the environmental risk of biochar and ENPs.

It is also worth emphasizing that multiple ENPs in different studies exhibit different mixture effects, since the mixture effects are commonly caused by the interaction of multiple factors. Thus, the toxicity of ENP mixtures can be reduced by modulating several controllable factors, such as changes in the chemical composition of the components present in the mixture, reduction of the effective exposure dose, and adjustment of the external environmental conditions. Note that abiotic field conditions can drive the transformation of ENPs in the natural environment, causing a reduction in the mixture effects of multiple ENPs. With respect to the mechanism of toxicity, it should be noted that the interaction of multiple ENPs with biological systems can cause different levels of damage, such as at the tissue level, organ level, cellular level, subcellular level, and biomolecular (glycans, lipids, proteins, and genes) level. In particular, the production of ROS can cause biomolecular damage and therefore excessive ROS production induced by multiple ENPs needs to be controlled by the organism. By optimizing the inherent structures and physicochemical properties of ENPs (e.g., size, purity, and surface properties), the direct interaction of ENPs with organisms and the uptake, accumulation, distribution, action, and clearance of ENPs in organisms can be improved. This also requires more purposely designed experiments investigating the impacts of the structure and properties of individual ENPs on the mixture effects induced by multiple ENPs.

### 3.5 Assessment and Prediction Methods for the Mixture Toxicity of Multiple ENPs

**Figure 3:**
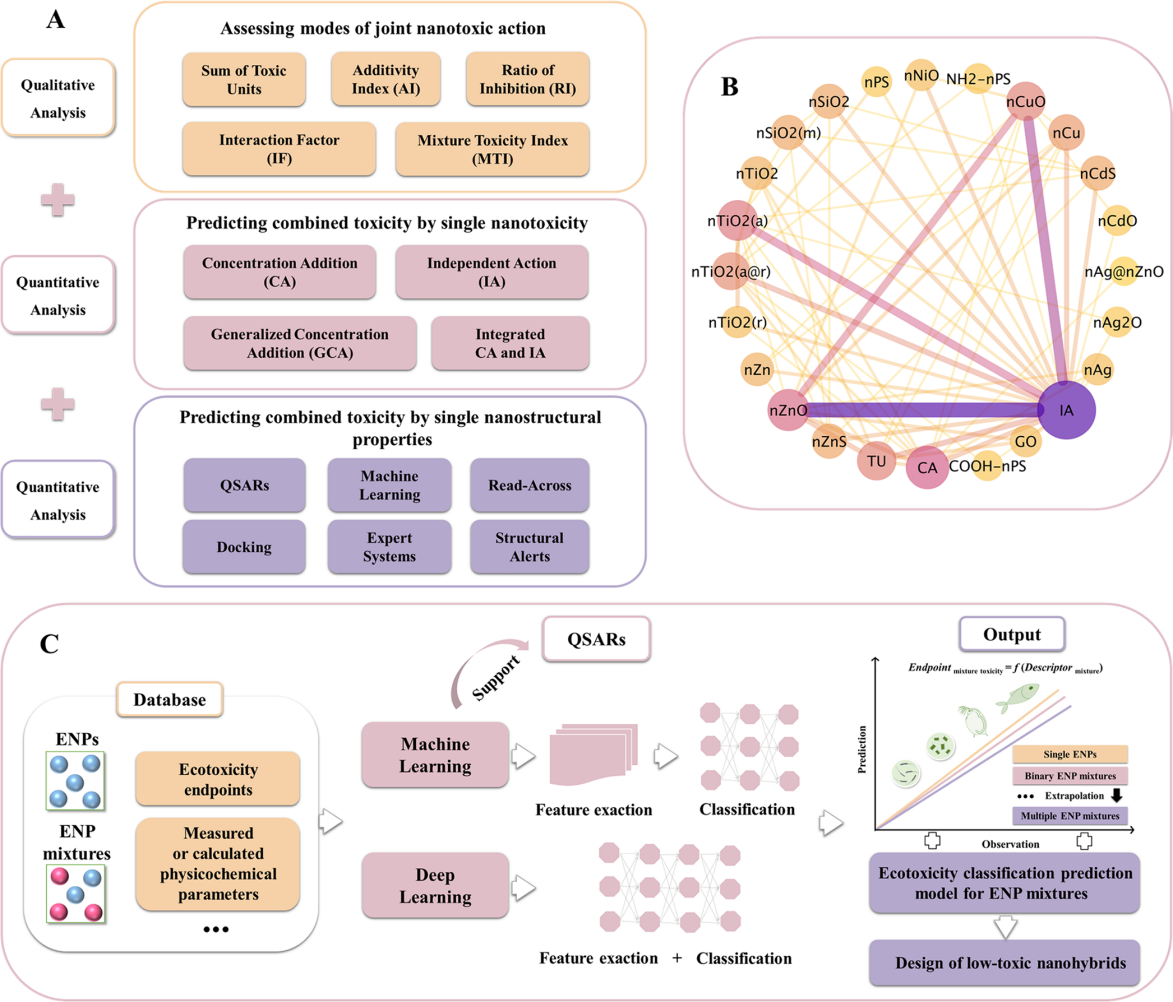
Assessment and prediction methodology of multi-ENP mixtures. (A) Schematic framework for the methodology. (B) Network diagram of association rules of ENPs in binary mixtures combined with the assessment methods for their joint toxicity (CA, concentration addition; IA, independent action; TU, toxic unit). (C) Scheme of machine-learning- or deep-learning-based QSAR approach used for the ecotoxicity prediction of the mixtures of individual ENPs.

Screening the risks of contaminants is mainly achieved by qualitatively assessing the types of joint interactions and quantitatively predicting the magnitude of mixture toxicity. Assessed and predictive methods (Figure 3A) may help to reduce the intensive laboratory experiments needed to determine the toxicity of mixtures of ENPs. An association analysis indicated that the most common way of assessing the joint interactions of multiple ENPs reported in existing studies is the IA-based model (Figure 3B). Moreover, the most frequently evaluated combination applying the IA-based method is the combination of nCuO and nZnO. Furthermore, it is estimated that the type of joint interaction of an ENP mixture is predicted correctly or overpredicted by default in approximately 42% of all combinations.

CA and IA models have been preliminarily applied to the assessment and prediction of the mixture toxicity of multiple ENPs. For example, Liu et al.^51^ applied CA and IA models to effectively predict the combined toxicity of nCu and nZnO to *Lactuca sativa* L., and the fit of the IA model to the experimental data on the combined toxicity of the two ENPs was higher than that of the CA model. Wang et al.^52^ used the IA model to effectively predict the combined toxicity of spherical nTiO_2_ and tubular nTiO_2_ to *C. pyrenoidosa*, while the CA model effectively predicted the combined toxicity of this binary mixture to *S. obliquus*. Although the CA and IA models offer some promise toward predicting the mixture toxicity of multiple ENPs, a great deal of validation will be necessary. In addition, one important realization is that the CA and IA models also require experiments to determine the toxicity characters (i.e., effect concentrations and concentration–response relationships) of all single components of a mixture. Taken together, the CA and IA models have become the two most commonly used methods in assessing and predicting the combined toxic effects of multiple ENPs, as shown in Figure 3B. Furthermore, the two methods are frequently used for the mixtures consisting of nCuO, nZnO, or nTiO_2_. In particular, toxicity assessment and prediction of mixtures containing nCuO and nZnO prefer IA models.

Quantitative structure activity relationship (QSAR) models are mathematical relationships between indicators of toxicity (e.g., lethality) and descriptors (e.g., physicochemical properties of chemicals).^78,79^ QSAR models have been successfully applied to predict the single toxicity of ENPs. However, the data that have been used for QSAR models were mostly generated from toxicity studies with single ENPs rather than making use of multiple ENPs. Currently, a limited number of studies have been developed to establish QSAR models for the photocatalytic activity and toxicity of nTiO_2_-based nanomixtures.^80−82^ These studies aimed to develop models for predicting the photocatalytic activity and cytotoxicity of nanoblends consisting of nTiO_2_ and (poly) metal clusters (Au, Ag, Pd, and Pt).^80−82^

QSAR models can fill in the limitations of CA and IA models.^83^ QSAR model inputs do not require the toxicity of all single components in a mixture or the dose–response curves of single components in the mixture. However, QSAR studies on the quantitative prediction of the mixture toxicity of multiple ENPs still constitute a knowledge gap. The main reason for this may be the lack of sufficient experimental data and the absence of uniform toxicity endpoints to develop predictive models. In addition to quantitative data on toxicity endpoints, descriptors are also important for the development of QSAR models. Descriptors for ENPs can be obtained based on the properties of nanoparticles at different scales,^84^ including physicochemical properties (e.g., chemical composition, shape, particle size, surface charge, specific surface area, and solubility), quantum chemical properties of nanocluster structures, and mesoscale nanoparticle properties. However, because ENP mixtures contain both nanoparticle and mixture components, there is a need to develop mixture descriptors for multiple ENPs and hence QSAR models can quantitatively predict the toxicity of multi-ENP mixtures. The weighted descriptor approach in eq 1 represents a preferred approach to developing descriptors for chemical mixtures (*D*_mix_).^85,86^ Then, a generic QSAR model for the prediction of activities of chemical mixtures can be expressed by eq 2(85)
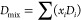
1
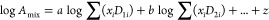
2where *A*_mix_ represents the activity of the chemical mixtures to be modeled, *x*_*i*_ represents the molar fraction of a component (*i*) in the mixtures, *D*_1_ and *D*_2_ are the structural descriptors used for each component, and *a*, *b*, and *z* are the coefficients of the regression function. A QSAR approach with mixture descriptors was implemented in a user-friendly application for assessing the aquatic toxicity of nanomixtures containing nTiO_2_ and one of the selected inorganic/organic compounds.^87^

Assessing and predicting the toxicity of mixtures of multiple ENPs is facing unprecedented opportunities and challenges. Computational nontesting methods (i.e., *in silico* models) representing a fast and reliable alternative approach to *in vivo* and *in vitro* methods, for example, machine learning, read-across, docking, expert systems, and structural alerts, are expected to play key roles in the toxicity prediction of mixtures of ENPs. In particular, the integration of QSAR and machine-learning methods (e.g., support vector machine, random forest, K-nearest neighbor, naïve Bayes, decision tree, neural network, and logistic regression) can serve as a very powerful tool for solving the problem of toxicity prediction of mixtures of NMs (Figure 3C). The reality, however, is that the lack of databases on the mixture toxicity of ENPs hinders the development and application of artificial-intelligence-based methods for toxicity prediction. As the size of the data increases, deep-learning methods perform better than machine-learning methods. It is worth noting that deep learning attempts to obtain high-level features directly from the data, which is the main difference between deep-learning and traditional machine-learning algorithms. In addition to the prediction of ecotoxicity endpoints/classification, machine-learning methods combined with QSAR notions can provide valuable hints for the design of low-toxicity nanohybrids. On balance, comprehensive and predictive knowledge about NM risks to environmental and ecological health must include explicit consideration of interactions in multiple ENP mixtures.

## 4 Outlook and Prospects

The mixture toxicity of multiple ENPs is an emerging topic, and this topic faces numerous opportunities and challenges. Based on the current state of the science, the following key research needs have emerged.

(1) Currently, single-component ENPs as the first generation have reached full market penetration. New-generation multicomponent NMs, made up of e.g. binary or ternary or quaternary constituents or ENP components with sometimes advanced properties, are just starting to enter the market. The association rule analysis performed shows that applying the notion of simple additivity is often justified, and the predictability of mixtures of ENPs can be done with approximately 42% accuracy by taking single ENP hazard information and using a simple additive approach. An understanding of joint interactions for those novel materials is in its infancy. Continued studies will be required to investigate the combined toxicity of hybrid NMs, particularly at environmentally relevant concentrations.
(2) Based on the single ENP data, the physicochemical behavior (e.g., stability, aggregation/agglomeration, dissolution) is the most important of all characteristics of ENPs. It is known that the presence of ligands to bind to and pH drive the single toxicity of ENPs. Thus, the effects of the physicochemical behavior such as stability (versus binding ligands) and pH versus dissolution on the toxicity of mixtures of ENPs need to be recognized. At the higher biological levels most experimental data collected for microbial communities and all other communities need to be estimated by making use of SSDs or other modeling techniques that are built from the standard laboratory test species data.
(3) When facing the continuous emergence of various new ENPs, the workload of the assessment and prediction of the mixture toxicity of multiple ENPs will multiply. In particular, the interaction behavior between different particles in the mixtures of ENPs has been screened but a mechanistic understanding has not been explored. In this study, we used the classical addition models and assumed antagonistic or synergistic joint interactions when a deviation on additivity was found. A 75% chance of a correct prediction would be given approximately when drawing lessons from making use of the CA and IA models for metal mixtures.^88−90^ The importance of modeling is recognized for screening purposes not only in prospective but also in retrospective effect assessments. Comprehensive computational approaches of predicting the mixture toxicity of multiple ENPs need to be developed further. This study gives the first building blocks on what data are currently present and accessible, and what types of joint interactions exist for mixtures of multiple ENPs and provides insights into what we can expect as response types for hybrid NMs.

## Supporting Information Available

Flowchart showing the decision process for inclusion and exclusion of literature on the ecotoxicity of mixtures of nanomaterials, identified using the ISI Web of Knowledge and PubMed search, list of studies on the joint toxicological effects of multiple metal-based engineered nanoparticles (ENPs) on ecological species, list of studies on the joint toxicological effects of multiple engineered nanoparticles (ENPs) comprised of nonmetal-based components on ecological species, list of studies on the potentiation or attenuation of effects of mixtures of individual engineered nanoparticles (ENPs) on ecological species, list of studies on the toxicological effects of multicomponent nanomaterials (NMs) on ecological species, and minimum inhibitory concentrations (MICs) for bacteria exposed to multicomponent nanomaterials (PDF)
