# Supplementary material for: Review and Prospects on the Ecotoxicity of Mixtures of Nanoparticles and Hybrid Nanomaterials
Source: Environ Sci Technol. 2022 Oct 5;56(22):15238–50. doi: 10.1021/acs.est.2c03333 (PMC9671040; doi:10.1021/acs.est.2c03333)
Supplement: Supplementary file 1 — es2c03333_si_001.pdf [file es2c03333_si_001.pdf]

Supporting Information

Review and prospects on the ecotoxicity of mixtures of nanoparticles and hybrid nanomaterials

Fan Zhang [a], Zhuang Wang [b], Willie J.G.M. Peijnenburg [a,c,*], Martina G. Vijver [a]

[a] Institute of Environmental Sciences (CML), Leiden University, Leiden 2300 RA, The Netherlands

[b] Collaborative Innovation Center of Atmospheric Environment and Equipment Technology, Jiangsu Key Laboratory of Atmospheric Environment Monitoring and Pollution Control, School of Environmental Science and Engineering, Nanjing University of Information Science and Technology, Nanjing 210044, People's Republic of China

[c] Centre for Safety of Substances and Products, National Institute of Public Health and the Environment (RIVM), Bilthoven 3720 BA, The Netherlands

* Corresponding author:

E-mail address: peijnenburg@cml.leidenuniv.nl (W. J. G. M. Peijnenburg)

Tables 4

Figures 2

Pages 37

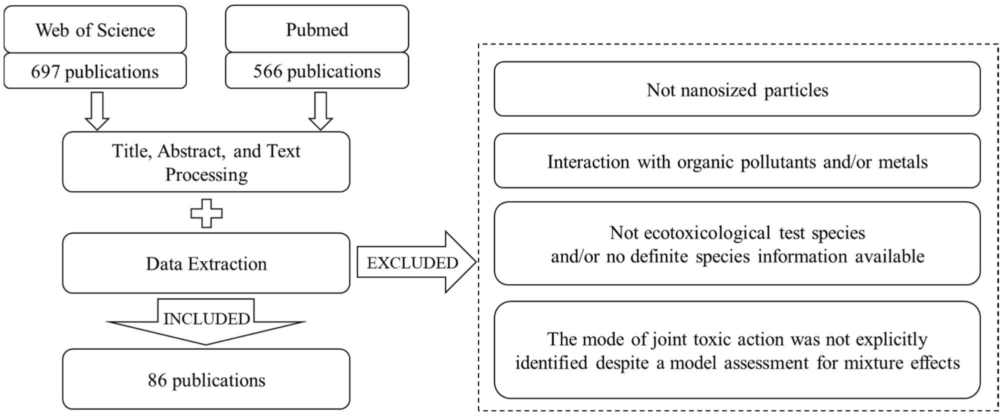

Figure S1. Flowchart showing the decision process for inclusion and exclusion of literature on the ecotoxicity of mixtures of nanomaterials, identified using the ISI Web of Knowledge and PubMed search.

Table S1. List of studies on the joint toxicological effects of multiple metal-based engineered nanoparticles (ENPs) on ecological species [a]

ENPs Types of mixtures	Ecological species	Test concentrations	Toxicity endpoints	Types of joint interactions	References
**Algae**					
$nTiO_2$ (anatase) + $nTiO_2$ (rutile)	*Chlorella* sp.	$nTiO_2$ (anatase) + $nTiO_2$ (rutile): 0.25+0.25, 0.25+0.5, and 0.5+0.5 mg/L	Cell viability, chlorophyll content, uptake/internalization, cell surface morphology, ultra-structural changes, DNA damage, and ROS generation	Antagonistic	Iswarya et al., 2015
		$nTiO_2$ (anatase) + $nTiO_2$ (rutile): 0.25+1, 0.5+0.25, 0.5+1, 1+0.25, 1+0.5, and 1+1 mg/L		Additive	
$nSiO_2$ + $nTiO_2$ (anatase@rutile)	*Scenedesmus obliquus*	$nSiO_2$: 1 μg/L and 1 mg/L<br>$nTiO_2$ (anatase@rutile): 1 μg/L and 1 mg/L<br>$nZrO_2$: 1 μg/L and 1 mg/L<br>Mixtures (1:1 and 1:1:1 ratios)	Chlorophyll content, intracellular levels of ROS, mitochondrial membrane potential, permeability of cell membrane, antioxidant activities, and cell surface morphology	n.d.	Liu et al., 2018
$nSiO_2$ + $nZrO_2$				n.d.	
$nTiO_2$ (anatase@rutile) + $nZrO_2$				n.d.	
$nSiO_2$ + $nTiO_2$ (anatase@rutile) + $nZrO_2$				Synergistic	
nCdS + nZnS	*Heterosigma akashiwo*	nCdS: 12 mg/L<br>$nSiO_2$ (with no inclusions): 143.5 mg/L<br>$nSiO_2$ (with metal inclusions): 2.1 mg/L<br>$nTiO_2$ (anatase): 79.5 mg/L<br>nZnS: 53 mg/L	Growth inhibition, esterase activity, membrane potential, ROS generation, and cell size	Antagonistic	Pikula et al., 2022
nCdS + $nTiO_2$ (anatase)				Synergistic	
nCdS+ $nSiO_2$ (with no inclusions)				Synergistic	
nCdS + $nSiO_2$ (with metal inclusions)				Antagonistic	

$nTiO_2$ (anatase) + nZnS				Synergistic	
$nSiO_2$ (with no inclusions) + nZnS				Synergistic	
$nSiO_2$ (with metal inclusions) + nZnS				Antagonistic	
$nSiO_2$ (with no inclusions) + $nTiO_2$ (anatase)				Synergistic	
$nSiO_2$ (with metal inclusions) + $nTiO_2$ (anatase)				Additive	
$nSiO_2$ (with no inclusions) + $nSiO_2$ (with metal inclusions)				Additive	
$nTiO_2$ (Spherical, anatase@rutile) + $nTiO_2$ (Tubular)	*Scenedesmus obliquus*	$nTiO_2$ (Spherical, anatase@rutile) + $nTiO_2$ (Tubular): 2.33+13.16 and 19.75+211.26 mg/L	Growth inhibition and intracellular ROS generation	Additive	Wang et al., 2020
	Chlorella pyrenoidosa	$nTiO_2$ (Spherical, anatase@rutile) + $nTiO_2$ (Tubular): 0.13+0.002 and 5.38+4.87 mg/L		Additive<br>Synergistic	
nCuO + nZnO	*Scenedesmus obliquus*	nCuO: 2.1 µg Cu/L-4.3 mg Cu/L<br>nZnO: 6.6 µg Zn/L-33.1 mg Zn/L<br>Mixtures: equal toxic ratio	Growth inhibition	Additive	Ye et al., 2017
**Bacteria**					
nAg + nPt	*Escherichia coli*<br>*Staphylococcus aureus*	nAg + nPt: 30+70, 50+50, and 70+30 wt%	Antimicrobial activity	n.d.	Breisch et al., 2020
	Escherichia coli			Synergistic	Chen et al., 2020

<table>
<tr><td>nCuO + nTiO$_2$ (anatase@rutile)</td><td></td><td>nCuO + nTiO$_2$ (anatase@rutile): 0.1+2, 0.2+2, 0.3+2, and 0.4+2 mg/L</td><td>Bacterial ATP levels, cell membrane integrity, and ROS production</td><td>Slight additive</td><td></td></tr>
<tr><td>nAg + nCuO</td><td rowspan="4">Nitrifying bacteria</td><td rowspan="4">The concentration of each metallic/oxide nanoparticles was 1 mg/L</td><td rowspan="4">Nitrification inhibition and intracellular ROS concentrations</td><td>Additive</td><td rowspan="4">Choi and Hu, 2009</td></tr>
<tr><td>nAg + nTiO$_2$ (anatase)</td><td>Additive</td></tr>
<tr><td>nAg + nZnO</td><td>Antagonistic</td></tr>
<tr><td>nAg + nCuO + nTiO$_2$ (anatase)</td><td>Additive</td></tr>
<tr><td>nTiO$_2$ (anatase) + nZnO</td><td>Escherichia coli</td><td>nTiO$_2$ (anatase):1, 10, 100, and 1000 mg/L
nZnO:1, 10, 100, and 1000 mg/L
Mixtures (1:1 ratio)</td><td>Growth reduction and cell wall damage</td><td>Antagonistic</td><td>Srivastava and Kumar, 2017</td></tr>
<tr><td rowspan="2">nTiO$_2$ (anatase@rutile) + nZnO</td><td>Escherichia coli</td><td rowspan="2">nTiO$_2$ (anatase@rutile) + nZnO: 10+1 and 10+25 mg/L</td><td rowspan="2">ATP levels, cell membrane integrity, ROS production, and nanoparticle/bacterial surface interactions</td><td rowspan="2">Antagonistic</td><td rowspan="2">Tong et al., 2015</td></tr>
<tr><td>Aeromonas hydrophila</td></tr>
<tr><td>nAg + nTiO$_2$ (anatase@rutile)</td><td>Escherichia coli</td><td>nAg: 5, 10, 20, 30, and 40 μg/L
nTiO$_2$ (anatase@rutile): 1 and 10 mg/L</td><td>ATP levels</td><td>n.d. (under dark)</td><td>Wilke et al., 2016</td></tr>
<tr><td>nAg + nTiO$_2$ (anatase@rutile)</td><td>Escherichia coli</td><td>nAg: 5, 10, 20, and 30 μg/L
nTiO$_2$ (anatase@rutile): 1 and 2 or 10 mg/L</td><td>ATP levels, cell membrane integrity, and ROS production</td><td>Synergistic (under light)</td><td>Wilke et al., 2018</td></tr>
<tr><td>nCeO$_2$ + nZnO</td><td rowspan="2">Nitrosomonas europaea</td><td>nCeO$_2$ + nZnO: 1+10, 10+10, and 50+10 mg/L</td><td rowspan="2">Cell size, charge, morphology, density, membrane integrity, ammonia removal rate, amoA gene expression, and AMO activity</td><td>Synergistic</td><td rowspan="2">Yu et al., 2016a</td></tr>
<tr><td>nCeO$_2$ + nTiO$_2$ (anatase)</td><td>nCeO$_2$ + nTiO$_2$ (anatase): 50+1, 50+10, and 50+50 mg/L</td><td>Antagonistic</td></tr>
</table>

$nTiO_2$ (anatase) + nZnO	*Nitrosomonas europaea*	$nTiO_2$ (anatase) + nZnO: 1+10, 10+10, and 50+10 mg/L	Cell size, charge, morphology, density, membrane integrity, ammonia removal rate, AMO activity, and transcriptional response	Antagonistic	Yu et al., 2016b
nAg + nCu	*Escherichia coli*	40 mL of nAg and 40 mL of nCu were separately synthesized in 3% (w/v) of chitosan and then mixed together	Bacterial growth inhibition	n.d.	Zain et al., 2014
	Bacillus subtilis				
nCuO + nZn	*Vibrio fischeri*	nCu (EC_{50}): 4.1 mg/L<br>nZn (EC_{50}): 20.5 mg/L<br>nCuO (EC_{50}): 118.7 mg/L<br>nZnO (EC_{50}): 11.6 mg/L<br>Equitoxic binary mixtures of nanoparticles were prepared based on the EC_{50} values of individual nanoparticles to determine their joint effects	Bioluminescence inhibition	Synergistic	Zhang et al., 2020
nCuO + nZnO				Synergistic	
nCu + nZn				Synergistic	
nCu + nCuO				Antagonistic	
nCu + nZnO				Antagonistic	
nZn + nZnO				Additive	
**Daphnia**					
nAg + nZnO	*Daphnia magna*	nAg: 0.05 to 0.25 mg·Ag/L and nZnO: 0.5 to 1.3 mg·Zn/L for immobilization tests; Combined exposures: based on a full factorial design<br>nAg: 0.095 to 0.5 mg·Ag/L and nZnO: 0.1 to 0.4 mg·Zn/L for reproduction tests; Combined exposures: a fixed ray design based on individual toxic units	Immobilization and reproduction	Synergistic	Azevedo et al., 2017
				Antagonistic	
$nTiO_2$ (anatase) + $nTiO_2$ (rutile)	*Ceriodaphnia dubia*	$nTiO_2$ (anatase): 4.63, 9.26, 13.89, 18.52, 23.15, 27.78, and 32.41 mg/L	Mortality and biouptake	Antagonistic (under	Iswarya et al., 2016

		$nTiO_2$ (rutile): 6, 12, 18, 24, 30, 36, and 42 mg/L<br>Mixtures: equal toxic proportions		visible irradiation)	
		$nTiO_2$ (anatase): 2.82, 5.64, 8.46, 11.28, 14.10, 16.92, and 19.74 mg/L<br>$nTiO_2$ (rutile): 2.97, 5.94, 8.91, 11.88, 14.85, 17.82, and 20.79 mg/L<br>Mixtures: equal toxic proportions		Additive (under UV-A irradiation)	
$nTiO_2$ (anatase) + $nTiO_2$ (rutile)	*Ceriodaphnia dubia*	Mixtures: 75, 300, and 1200 μM<br>the mixtures treated algal diet<br>In case of a binary mixture, the equal concentration of anatase and rutile nanoparticles forms the total concentration of binary mixture	Mortality, ultra-structural deformities, bioaccumulation, and biomagnification	Antagonistic (under visible irradiation)<br>Antagonistic (under UV-A irradiation)	Iswarya et al., 2018
$nTiO_2$ (anatase) + $nTiO_2$ (rutile)	*Ceriodaphnia dubia*	Mixtures: 75, 150, 300, 600, and 1200 μM<br>the mixtures treated algal diet<br>The binary mixture comprises an equal concentration of rutile and anatase nanoparticles	Mortality and oxidative stress (MDA, CAT, and GSH)	Synergistic (lower concentration, under visible irradiation)<br>Additive (higher concentration, under visible irradiation)<br>Additive (lower concentratio	Iswarya et al., 2019

				n, under UV-A irradiation)<br>Antagonistic (higher concentration, under UV-A irradiation)	
nAg + nZnO	*Daphnia magna*	nAg: 1-25 µg/L and nZnO: 0.25-5 mg/L	Immobilization and feeding inhibition	Synergistic	Lopes et al., 2016
nCu + nCr	*Daphnia magna*	Joint toxicity of binary mixtures was determined at an equal concentration (1:1), and the total concentrations were 0.4, 2, 10, 50, and 100 µg/L	Reproduction and growth, rates of filtration and ingestion, as well as changes in enzyme activities: AChE, SOD, CAT, and GST	More-than-additive	Lu et al., 2017
$nTiO_2$ (anatase) + $nTiO_2$ (rutile)	*Daphnia similis*	70:30 anatase: rutile ratio (w/w)<br>1 to 100 mg/L TiO_2	Immobilization	n.d.	Marcone et al., 2012
nCu + nZnO	*Daphnia magna*	nCu + nZnO: 0.11 mg Cu/L+1.29 mg Zn/L<br>nCu + nZnO: 0.40 mg Cu/L+4.01 mg Zn/L	Mortality and bioaccumulation	Additive<br>More-than-additive	Yu et al., 2022
nCuO + nZnO	*Daphnia magna*	Binary mixtures were also tested according to an equiconcentration ratio of 1:1 and the total exposure concentrations were 0.0004, 0.002, 0.01, 0.05, and 0.25 mg/L	Immobilization, mortality, reproduction (fecundity) and growth, as well as filtration and ingestion rates	Synergistic<br>Partial additive	Zhao et al., 2012
**Fish**					
	Cyprinus carpio			Antagonistic	

nAg + $nTiO_2$ (anatase@rutile)		nAg: 0.05, 0.10, 0.20, 0.30, 0.40, 0.50, 0.60, and 0.70 mg/L for acute toxicity tests and nAg: 0.05 and 0.1 mg/L for chronic toxicity tests<br>$nTiO_2$ (anatase@rutile): 1 mg/L	Mortality, bioaccumulation, oxidative stress (SOD, CAT, and GST), and gill histopathology	Synergistic<br>Additive	Haghighat et al., 2021
nCu + nZnO	*Poeciliopsis lucida*	nCu: 0.39, 0.78, 1.56, 3.13, 6.25, 12.5, and 25 µg/mL<br>nZnO: 6.25 µg/mL	Cell viability, cell morphology, and metal internalization	n.d.	Hernández-Moreno et al., 2016
nCu + nZnO	*Oncorhynchus mykiss*	nCu: 0.0425, 0.085, 0.17, and 0.34 mg/L<br>nZnO: 1.25 mg/L	Survival, metal internalization, and oxidative stress (EROD activity, GST activity, and GSH/GSSG ratio)	n.d.	Hernández-Moreno et al., 2019
$nTiO_2$ (spherical, anatase) + nZnO (stick-shaped)	*Danio rerio*	$nTiO_2$ (spherical, anatase): 1.5, 3, 6, 12, and 24 mg Ti/L<br>nZnO: 2, 4, 8, 16, and 32 mg Zn/L	Mortality and hatching rate	Antagonistic	Hua et al., 2016
$nCeO_2$ + nCuO	Zebrafish embryos	$nCeO_2$: 0.01, 0.1, 1, 10, and 50 µg/mL<br>nCuO: 0.01, 0.1, 1, 10, and 50 µg/mL<br>Mixtures (1:1 ratio)	Mortality rate, hatching rate, malformations, oxidative stress genes, CAT enzyme activity, DNA damage, and apoptosis and necrosis	n.d.	Kaur et al., 2019
nCuO + $nTiO_2$ (anatase@rutile)	*Cyprinus carpio*	nCuO + $nTiO_2$ (anatase@rutile): 2.5+10 and 5.0+10 mg/L	Oxidative stress biomarkers<br>in the liver, brain, and gills and acetylcholinesterase activity (a biomarker that indicates neurotoxicity) in the brain and muscle, as	n.d.	Mansouri et al., 2016

			well as induce histopathological alterations in the gills, liver and retina		
$nCuO + nTiO_2$ (anatase@rutile)	*Cyprinus carpio*	$nCuO + nTiO_2$ (anatase@rutile): 2.5+10 and 5.0+10 mg/L	Histopathological anomalies of gill and intestine tissues in *C. carpio*	Synergistic	Mansouri et al., 2017
$nTiO_2 + nZnO$	*Prochilodus lineatus*	$nTiO_2 + nZnO$: 1+1 μg/L	Biochemical responses (AchE activity, protein carbonylation, lipid peroxidation, and non-protein thiols) and injuries in organs (histological and ultra-structural analyses)	n.d.	Miranda et al., 2016
nAg + nCuO	*Clarias gariepinus*	nCuO: 6.25, 12.5, 25, 50, and 100 mg/L<br>nAg: 6.25, 12.5, 25, 50, and 100 mg/L<br>Mixtures (1:1 ratio)	Frequency of micronucleus, haematology, histopathology (skin, gills and liver), and hepatic oxidative stress analysis (MDA, reduced GSH, SOD, and CAT)	Antagonistic<br>Synergistic	Ogunsuyi et al., 2019
$nCuO + nCeO_2$	*Carassius auratus*	20, 40, 80, 160, and 320 mg/L. The binary and ternary mixtures were tested at an equi-concentration ratio of 1:1 or 1:1:1 (W/V)	AChE activity, Na^+/K^+-ATPase activity, SOD activity, and CAT activity	Antagonistic	Xia et al., 2013
$nCuO + nZnO$				Synergistic	
$nCeO_2 + nZnO$				Antagonistic	
$nCeO_2 + nCuO + nZnO$				Additive	
**Fungi**					

nAg + $nMoS_2$ (chitosan functionalization)	*Saccharomyces cerevisiae*	nAg: 5, 10, 20, 30, and 40 µg/L<br>$nMoS_2$ (chitosan functionalization): 1 and 10 mg/L	Oxidative stress (intracellular ROS generation), membrane stress (intracellular lactate dehydrogenase activity), and metabolic activities	Synergistic	Yang et al., 2018
**Insects**					
nCdO + nPbO	*Apis mellifera*	nCdO: 0.01 mg/mL<br>nPbO: 0.65 mg/mL	Content of nCdO and nPbO in midgut tissues, survival, morphological assessment of midgut tissues, ultrastructure observations, and incidence of apoptosis and necrosis of midgut epithelia	Antagonistic	Dabour et al., 2019
nZn + nCu	*Folsomia candida*	nZn: nCu: 300+300 mg/kg	Survival and reproduction	Antagonistic	Jośko et al., 2022
nZnO + nCuO	*Folsomia candida*	nZnO: nCuO: 300+300 mg/kg	Survival and reproduction	Synergistic	Jośko et al., 2022
**Plants**					
nCo + nFe + nNi	*Lactuca sativa*	Influent: 2,700 mg nCo + 50,000 mg nFe + 6,250 mg nNi; DI Water 123 kg	Germination and growth	n.d.	Hassanein et al., 2021
$nTiO_2$ (anatase) + nZnO	*Vigna angularis*	$nTiO_2$ (anatase): 20, 40, 60, 80, 100, and 200 µg/mL<br>nZnO: 20, 40, 60, 80, 100, and 200 µg/mL<br>Mixtures (1:1 ratio)	Seed germination, root/shoot length, total chlorophyll content, carotenoids and lipid peroxidation, oxidative stress and antioxidant enzyme activity, kinetic uptake and transport	n.d.	Jahan et al., 2018
nCuO + nZnO	*Hordeum vulgare*	nCuO: 300 mg Cu/kg	Biomass, plant mineral	n.d.	Jośko et al., 2021

		nZnO: 300 mg Zn/kg<br>Mixtures (1:1 ratio)	composition as well as expression of genes regulating metal homeostasis (ZIP1,3,6,8,10,14, RAN1, PAA1,2, MTP1, COPT5) and detoxification (MT1–3)		
nCuO + nZnO<br>nCuO + $nTiO_2$<br>nCuO + nCr_2O_3<br>nCuO + nFe_2O_3<br>nZnO + $nTiO_2$<br>nZnO + nCr_2O_3<br>nZnO + nFe_2O_3	*Lepidium sativum*<br>*Linum usitatissimum*<br>*Cucumis sativus*<br>*Triticum aestivum*	Concentration of each nanoparticles was set to be 100 mg/L<br>Mixtures (1:1 ratio)	Seed germination, root growth inhibition rates, and the external and internal surface area of root	Antagonistic	Jośko et al., 2017
nCdO + nCuO	*Vigna radiata*	0.1, 1, and 10 mg/L<br>Mixtures (1:1 ratio)	Germination percent, relative germination rate, and metal accumulations	n.d.	Jung et al., 2020
nCuO + nZnO<br>nCuO + nNiO<br>nZnO + nNiO	*Lactuca sativa*	nCuO: 0.06 and 0.12 mg/L<br>nZnO: 0.12 and 0.25 mg/L<br>nNiO: 0.15 and 0.3 mg/L	Root and shoot growth	Additive	Kong et al., 2021
	Raphanus sativus	nCuO: 0.09 and 0.18 mg/L<br>nZnO: 0.31 and 0.62 mg/L<br>nNiO: 0.71 and 1.42 mg/L			
nCu + nZnO	*Lactuca sativa*	nCu: 0.10 to 0.80 mg/L<br>nZnO: 0.50 to 50.00 mg/L	Relative root elongation rate	Antagonistic	Liu et al., 2016
$nTiO_2$ (anatase) + $nTiO_2$ (rutile)	*Pisum sativum*	800 mg of TiO_2 per kg of soil<br>Mixture of anatase and rutile $nTiO_2$: 1:1 ratio	TiO_2 particles' entry in the root system,	n.d.	Muccifora et al., 2021

			bioaccumulation, relative distribution, and localization, as well as the main crystalline form preferentially absorbed and their effect in cells ultrastructure of plant roots		
nCuO + nZnO	*Spinacia oleracea*	nCuO: 10, 100, and 1000 mg/L<br>nZnO: 10, 100, and 1000 mg/L<br>Mixtures (1:1 ratio)	Root length, shoot length, total weight, chlorophyll content, carotenoid content, and ion content of *S. oleracea* plants	n.d.	Singh and Kumar, 2016
nCuO + nZnO	*Raphanus sativus*	nCuO: 10, 100, and 1000 mg/kg<br>nZnO: 10, 100, and 1000 mg/kg<br>Mixtures (1:1 ratio)	Seed germination (root length, shoot length, and fresh weight) and metal uptake	Antagonistic	Singh and Kumar, 2018
nCuO + nZnO	*Raphanus sativus*	nCuO: 0.1, 1, 10, 100, and 1000 mg/L<br>nZnO: 0.1, 1, 10, 100, and 1000 mg/L<br>Mixtures (1:1 ratio)	Seed germination (root length, shoot length, and fresh weight) and metal uptake	Antagonistic	Singh and Kumar, 2019
nCuO + nZnO	*Spinacia oleracea*	nCuO + nZnO: $1.2\times10^{-4}+1.2\times10^{-4}$, $1.2\times10^{-3}+1.2\times10^{-3}$, $1.2\times10^{-2}+1.2\times10^{-2}$ mol/kg of soil	Maturity, plant fresh weight, root length, and metal uptake	Additive	Singh and Kumar, 2020a
nAg_2O + $nTiO_2$ (anatase)	*Spinacia oleracea*	nAg_2O: 1 and 10 mg/kg<br>$nTiO_2$ (anatase): 1 and 10 mg/kg<br>Mixtures (1:1 ratio)	Plant physiology and development (root length, shoot length, and fresh weight), total chlorophyll and carotenoid contents, and metal uptake	Additive	Singh and Kumar, 2020b

$nCeO_2$ + nZnO	*Pisum sativum*	Ce: 100 and 200 mg/L<br>Zn: 100 and 200 mg/L<br>Mixtures (1:1 ratio)	Plant growth (root and stem lengths and fresh weight), Ce and Zn concentrations in roots and shoots, photosynthesis pigments (contents of chlorophyll a, chlorophyll b, and carotenoids), and photosynthetic parameters (leaf net photosynthesis, sub-stomatal CO_2 concentration, transpiration, stomatal conductance, photosynthetic water use efficiency, and photosynthetic CO_2 response curve	n.d.	Skiba et al., 2021
nCdO + nCuO	*Vigna radiata*	nCdO + nCuO: 1+1, 10+10, and 100+100 mg/kg	Seed germination, plant growth, and metal accumulation	Antagonistic	Subpiramaniyam et al., 2021

[a] N.d. = not determined. AChE – acetylcholinesterase, AMO – ammonia monooxygenase, ATP – adenosine triphosphate, ATPase – adenosine triphosphatase, CAT – catalase, COX – cyclooxygenase, EROD – ethoxyresorufin-O-deethylase, GSH – glutathione, GSSG – oxidized glutathione, GST – glutathione S-transferase, LPO – lipid peroxidation, MDA malondialdehyde, $nMoS_2$ – molybdenum disulfide nanosheets, ROS – reactive oxygen species, SOD – superoxide dismutase.

For presentation purposes, $nSiO_2$ (with metal inclusions) is shortened to $nSiO_2$(m), $nTiO_2$ (anatase) is shortened to $nTiO_2$(a), $nTiO_2$ (anatase@rutile) is shortened to $nTiO_2$(a@r), $nTiO_2$ (rutile) is shortened to $nTiO_2$(r).

Table S2. List of studies on the joint toxicological effects of multiple engineered nanoparticles (ENPs) comprising of nonmetal-based components on ecological species [a]

**ENPs<br>Types of mixtures**	**Ecological species**	**Test concentrations**	**Toxicity endpoints**	**Types of joint interactions**	**References**
nPS + $nTiO_2$ (anatase@rutile)	*Scenedesmus obliquus*	nPS: 1 mg/L<br>$nTiO_2$ (anatase@rutile): 0.025, 0.25, and 2.5 mg/L	Cell viability, morphological changes, oxidative stress (total ROS, superoxide radical, hydroxyl radical), antioxidant activity, photosynthetic efficiency, and esterase activity	Antagonistic<br>Additive	Das et al., 2022
nPS + nZnO	*Ctenopharyngodon idella*	nPS: 760 µg/L<br>nZnO: 760 µg/L	Behavioral, biochemical (nitric oxide dosage, TBARS, hydrogen peroxide, total glutathione content, DPPH radicals' scavenging, SOD, and AChE activity, nutritional status), and genotoxic biomarkers	No observed antagonistic, synergistic or additive effect	Estrela et al., 2021
MWCNTs + nCuO	*Tetradesmus obliquus*	MWCNTs: 1, 10, and 100 mg/L<br>nCuO: 2 and 200 mg/L	Growth inhibition, membrane damage, physical damage, oxidative stress (ROS level, SOD, and MDA), and internalization of Cu	n.d.	Fang et al., 2022
nSe + nZnO	Zebra fish *(D. rerio)*	nSe + nZnO (2 mg/kg each)	Survivability, growth performance parameters, intracellular ROS, gene expression, and fecundity and development	Synergetic	Fasil et al., 2021

MWCNTs + nZnO	*Brassica rapa*	MWCNTs: 10 and 100 mg/L<br>nZnO: 10, 50, and 100 mg/L	The length of roots and stems, chlorophyll content, oxidative stress (relative ROS, soluble sugar, and MDA contents), antioxidant enzyme activity (CAT, POD, and SOD), metal element content, and root scanning electron microscopy	Synergetic	Hong et al., 2022
nPS + nAg	*Chlamydomonas reinhardtii*	nAg: 3, 10, 30, 100, and 200 μg/L<br>nPS: 3 and 30 mg C/L	Cell-specific growth rate and subcellular distributions	Synergistic	Huang et al., 2019
	Ochromonas danica	nAg: 10, 30, 100, 200, and 300 μg/L<br>nPS: 3 and 30 mg C/L			
nPS + $nTiO_2$ (anatase@rutile)	*Chlorella* sp.	nPS, COOH-nPS, and NH_2-nPS: 5 mg/L<br>$nTiO_2$ (anatase@rutile): 0.25, 0.5, and 1 mg/L	Cell viability, oxidative stress (total ROS, superoxide and hydroxyl radical, CAT and SOD, and MDA), maximum quantum yield of PS II, and esterase activity	Antagonistic	Natarajan et al., 2022
COOH-nPS + $nTiO_2$ (anatase@rutile)					
NH_2-nPS + $nTiO_2$ (anatase@rutile)					
GNs + nZnO	*Capoeta fusca*	GNs + nZnO: 6.5+0.04 and 6.5+0.09 mg/L	Bioconcentration (uptake and elimination)	n.d.	Sayadi et al., 2021
MLGs + nZnO	*Capoeta fusca*	MLGs: 6.5 mg/L<br>nZnO: 0.1, 0.4, 0.9, 1, 5, 10, 15, 20, 25, and 30 mg/L for acute toxicity test and nZnO: 0.09 mg/L for behavioural assay and histopathology	Lethality, histopathological and behavioral changes	Synergistic	Sayadi et al., 2022
				Antagonistic	
GO + nZnO	*Scenedesmus obliquus*	GO: 0.5-50 mg/L<br>nZnO: 0.01-50 mg/L<br>Mixture ratios: EC_{10} and EC_{50} of each component	Growth inhibition rate and total ROS level	Additive	Ye et al., 2018

	Daphnia magna	GO: 1-80 mg/L<br>nZnO: 0.01-0.4 mg/L<br>Mixture ratios: EC_{10} and EC_{50} of each component	Immobilization rate and total ROS level	Additive	
	Danio rerio	GO: 20-160 mg/L<br>nZnO: 2-20 mg/L<br>Mixture ratios: LC_{10} and LC_{50} of each component	Lethality and total ROS level	Antagonistic	
CNCs + nZnO	*Eremosphaera viridis*	CNCs: 100 mg/L<br>nZnO: 1, 5, and 10 mg/L	Dry weight, chlorophyll a, chlorophyll b, ROS level, CAT activity, MDA content, cellular superficial- and ultra-structures, elemental distribution as well as proteins and lipids in a single algal cell	n.d.	Yin et al., 2022
GNs + $nZrO_2$	*Chlorella pyrenoidosa*	GNs: 0.1 and 1 mg/L<br>$nZrO_2$: 1, 5, 10, 17.5, 25, and 50 mg/L<br>GNs + $nZrO_2$: 1+EC_{10} and 1+EC_{50} mg/L	Growth inhibition, intracellular levels of ROS, mitochondrial membrane potential, permeability of cell membrane, and cellular superficial- and ultra-structures	Synergistic	Wang et al., 2021
rGO + $nZrO_2$		rGO: 0.1 and 1 mg/L<br>$nZrO_2$: 1, 5, 10, 17.5, 25, and 50 mg/L<br>rGO + $nZrO_2$: 1+EC_{10} and 1+EC_{50} mg/L		Synergistic	
MWCNTs + nPS	*Microcystis aeruginosa*	MWCNTs: 5, 10, 20, and 50 mg/L<br>nPS: 5, 10, 20, and 50 mg/L	Growth (cell density), photosynthesis (chlorophyll a), total protein, antioxidant responses (SOD and MDA), membrane damage, genetic material damage, and metabolic process	Antagonistic	Zhang et al., 2022

GO + nAl_2O_3	*Chlorella pyrenoidosa*	GO: 25 mg/L<br>nAl_2O_3: 50, 100, 150, 300, 450, and 600 mg/L	Growth inhibition, membrane damage, oxidative stress, and physical damage	n.d.	Zhao et al., 2018
GQDs + nZnO	*Gymnodinium*	GQDs + nZnO: 1+1, 20+5, and 20+20 mg/L	Cell density, specific growth rates, total intracellular ROS, enzyme activities (SOD and ATPase), and surface interaction of nanoparticles and algal cells	Antagonistic	Zhu et al., 2022

[a] N.d. = not determined. AChE – acetylcholinesterase, ATPase – adenosine triphosphatase, CNCs – cellulose nanocrystals, COOH-nPS – carboxyl-functionalized polystyrene nanoplastics, DPPH – diphenyl-1-picrylhydrazyl, EC_{10} – 10% effect concentration, EC_{50} – 50% effect concentration, GNs – graphene nanosheets, GO – graphene oxide, GQDs – graphene quantum dots, LC_{10} – 10% lethal concentration, LC_{50} – 50% lethal concentration, MDA– malondialdehyde, MLGs – multi-layer graphenes, MWCNTs – multiwall carbon nanotubes, NH_2-nPS – amine-functionalized polystyrene nanoplastics, POD – peroxidase, nPS – polystyrene nanoplastics, rGO – reduced graphene oxide, nSe – nano-selenium, SOD – superoxide dismutase, SWCNTs – single walled carbon nanotubes, TBARS – thiobarbituric acid reactive species.
For presentation purposes, $nTiO_2$ (anatase@rutile) is shortened to $nTiO_2$(a@r).

Table S3. List of studies on the potentiation or attenuation of effects of mixtures of individual engineered nanoparticles (ENPs) on ecological species [a]

ENPs Types of mixtures	Ecological species	Potentiation or attenuation of effects		References
nAg + nPt	*Escherichia coli*	nPt significantly increased the toxicity of nAg	↑	Breisch et al., 2020
	Staphylococcus aureus			
MWCNTs + nCuO	*Tetradesmus obliquus*	The existence of nCuO in some groups reduced cell membrane damage caused by MWCNTs	↓	Fang et al., 2022
		The highest concentration of nCuO combined with the highest concentration of MWCNTs enhanced the induced ROS level	↑	
nAg + $nTiO_2$ (anatase@rutile)	*Cyprinus carpio*	$nTiO_2$ increased acute toxicity of nAg	↑	Haghighat et al., 2021
		$nTiO_2$ increased Ag accumulation in liver and intestine	↑	
		$nTiO_2$ decreased Ag accumulation in gills	↓	
		$nTiO_2$ somewhat mitigated the effects of nAg on antioxidant enzymes activities	↓	
nCu + nZnO	*Poeciliopsis lucida*	The cytotoxicity exerted by nCu was enhanced in presence of non-toxic concentrations of nZnO	↑	Hernández-Moreno et al., 2016
nCu + nZnO	*Oncorhynchus mykiss*	The co-exposure of rainbow trout to non-toxic concentrations of nCu and a fixed non-toxic concentration of nZnO resulted in lethal effects	↑	Hernández-Moreno et al., 2019
$nTiO_2$ (anatase) + nZnO	*Vigna angularis*	The combination led to attenuated uptake and translocation behavior	↓	Jahan et al., 2018
nCuO + nZnO	*Hordeum vulgare*	After combined treatment of ENPs, the extractable concentrations of Cu and Zn were lower than upon individual exposure in bulk soil	↓	Jośko et al., 2021
		Genes related to metal uptake (ZIP) and cellular compartment (PAA2, RAN1) were mostly up-regulated by single rather than combined application of ENPs	↓	
nCdO + nCuO	*Vigna radiata*	The germination rate of the nCdO + nCuO treatment was less than that of the single metal exposure under both humidities (70% and 80%) at 48 h	↓	Jung et al., 2020

$nCuO + nCeO_2$	Zebrafish embryos	The harmful effects of the mixtures were more than $nCeO_2$ and less than that of nCuO	↑↓	Kaur et al., 2019
$nCuO + nTiO_2$ (anatase@rutile)	*Cyprinus carpio*	The joint presence of $nTiO_2$ can potentially increase the uptake of nCuO in the tissues of carp	↑	Mansouri et al., 2016
$nCeO_2 + nZnO$	*Pisum sativum*	The effects of nZnO were decreased by $nCeO_2$	↓	Skiba et al., 2021
GNs + nZnO	*Capoeta fusca*	The presence of GNs reduced the bioavailability of nZnO	↓	Sayadi et al., 2021
$nAg + nTiO_2$ (anatase@rutile)	*Escherichia coli*	$nTiO_2$ attenuated the toxicity of nAg	↓	Wilke et al., 2016
$nAg + nMoS_2$ (chitosan functionalization)	*Saccharomyces cerevisiae*	$nMoS_2$ attenuated the oxidative stress induced by nAg on the yeast cells	↓	Yang et al., 2018
		nAg inhibited the metabolic activities in yeast cells, but this inhibition phenomenon could be alleviated by $nMoS_2$	↓	
CNCs + nZnO	*Eremosphaera viridis*	The addition of CNCs enhanced the bioavailability and toxicity of nZnO to the algae	↑	Yin et al., 2022
		The nZnO-CNC association enhanced the envelopment of the algal cells and exerted strong oxidative stress as compared to bare nZnO	↑	
$GO + nAl_2O_3$	*Chlorella pyrenoidosa*	Algal growth inhibition by GO with coexisting nAl_2O_3 particles was much lower than the sum of inhibitions from the individual materials for nAl_2O_3, showing the toxicity mitigation by nAl_2O_3	↓	Zhao et al., 2018
		GO-induced algal membrane damage was suppressed by the nAl_2O_3	↓	

[a] ↑ indicates the potentiation of effect of mixtures of individual ENPs and ↓ indicates the attenuation of effect of mixtures of individual ENPs. CNCs – cellulose nanocrystals, GNs – graphene nanosheets, GO – graphene oxide, MWCNTs – multiwall carbon nanotubes.
For presentation purposes, $nTiO_2$ (anatase) is shortened to $nTiO_2$(a), $nTiO_2$ (anatase@rutile) is shortened to $nTiO_2$(a@r).

Table S4. List of studies on the toxicological effects of multicomponent nanomaterials (NMs) on ecological species [a]

Types of hybrid NMs	Ecological species	Toxicity endpoints	Minimum inhibitory concentration	Toxic effects	References
nAg@nZnO	*Daphnia magna*	Immobilization and reproduction	n.d.	nAg@nZnO hybrid NMs showed higher toxicity than predicted based on the toxicity of nAg and nZnO	Azevedo et al., 2017
GO@nZnO	*Escherichia coli*	Growth of bacteria	n.d.	The antibacterial activity of GO@nZnO nanorods hybrid NMs has been demonstrated	Bhaisare et al., 2016
	Staphylococcus aureus				
α-nFe_2O_3@nCo_3O_4	*B. subtilis*	Bacterial growth inhibition	90 mg/dL	The enhanced bactericidal activity of the α-nFe_2O_3@nCo_3O_4 nanocomposite was the result of synergistic effects of iron oxide and cobalt oxide nanoparticles	Bhushan et al., 2018
	S. aureus		75 mg/dL		
	E. coli		60 mg/dL		
	S. typhi		45 mg/dL		
GO@nAg	*Fusarium graminearum*	Spore germination inhibition	n.d.	The GO@nAg nanocomposite showed almost a 3- and 7-fold increase of inhibition efficiency over pure nAg and GO suspension, respectively.	Chen et al., 2016
$nTiO_2$@MWCNT	*Danio rerio* embryos	Acute toxicity, hatching rate, growth, yolk sac size, and sarcomere length	n.d.	TiO_2@MWCNT hybrid NMs showed no acute toxicity to zebrafish embryos	Da Silva et al., 2018
GO@nAg	Zebrafish embryos	Mortality, malformation, edema, hatching, total length, and yolk sac size	n.d.	With chorion: LC_{50} of GO@nAg hybrid NMs: 1.4 [1.3-1.7] mg/L; Without chorion: LC_{50} of GO@nAg hybrid NMs: 1.0 [0.9-1.2] mg/L; The toxic effects of GO@nAg were lower than $AgNO_3$, but higher than GO	de Medeiros et al., 2021

nSe@nIO	*Staphylococcus aureus*	Biofilm viability	n.d.	The relative fraction of dead-to-live bacteria of the nanocomposites (400.0%) was much higher than that of nSe (51.6%) and nIO (60.0%)	Li et al., 2020
GO@polyvinylpyrrolidone-stabilized nAg	*Pseudomonas aeruginosa*	Bacterial growth inhibition	n.d.	This hybrid nanocomposite poses enhanced antibacterial activity against carbapenem-resistant *P. aeruginosa* strains through a possible synergy between toxicity mechanisms of GO nanosheets and nAg	Lozovskis et al., 2020
nTiO_2@MWCNT-CNF	*Pseudokirchneriella subcapitata*	Growth inhibition and sublethal oxidative stress	n.d.	Acute exposure of *P. subcapitata* to various concentrations of TiO_2@MWCNT-CNF nanocomposite may cause algal growth inhibition including undesirable sublethal oxidative stress effects	Malatjie et al., 2022
nZn@nCuO	*Xenopus laevis* embyos	Bioaccumulation, oxidative stress, and histopathology	n.d.	nZn@nCuO nanocomposite does induce only mild acute toxicity in *X. laevis* embryos. Nevertheless, these effects are smaller than those of nZnO. Interestingly, embryos exposed to the nanocomposite accumulate NPs more efficiently than those exposed to nCuO and nZnO, but the internalized NMs do not induce severe acute toxicity	Mantecca et al., 2015
nAg@GO<br>Chit-nAg@GO	*Staphylococcus aureus* UCLA 8076<br>*Staphylococcus aureus* 1190R	Bacterial growth inhibition	nAg@GO: 1.90 Ag + 1.5 GO µg/mL<br>Chit-nAg@GO (1:8): 1.19 Ag + 1.41 GO µg/mL	Chit-nAg@GO exhibit higher antibacterial activity than most of the antibacterial agents based on nAg or nAg@GO reported	Marta et al., 2015

PSF-CNF@nAg	*Bacillus subtilis*	Bacterial growth inhibition	n.d.	In solid phase the gram-positive bacteria showed higher sensitivity for PSF-CNF@nAg membranes, while in liquid phase the antimicrobial activity of the hybrid membrane is more pronounced towards gram-negative species. Furthermore, in the case of *E. coli*, the growth inhibition in liquid medium is probably due to the synergetic action of the modified CNF and nAg	Mocanu et al., 2019
	Escherichia coli				
Ag-nZnO@SWCNT	*Escherichia coli*	Viable cell numbers	n.d.	All multicomponent NMs have been reported to possess strong antimicrobial activity towards *E. coli* and *S. aureus* bacteria, due to synergistic effect between metal-doped ZnO nanoparticles and carbon nanotubes	Mohammed et al., 2019
Au-nZnO@SWCNT					
Ag-nZnO@MWCNT	*Staphylococcus aureus*				
Au-nZnO@MWCNT					
nAg@GO	*Escherichia coli*	Antimicrobial effect mean inhibition zone	n.d.	An increase in the inhibition zone with the increase in amount of nAg@GO nanocomposite is obvious due to greater antimicrobial agents	Naeem et al., 2019
	Staphylococcus aureus				
rGO@nCu$_2$O	*Escherichia coli*	Bacterial growth inhibition	5.9 µg/mL	rGO@nCu$_2$O nanocomposite have a higher antimicrobial activity toward gram-negative and gram-positive bacteria when compared with reference antibiotics such as kanamycin and streptomycin	Selim et al., 2020
	Pseudo-monas aeruginosa		2.9 µg/mL		
	Bacillus subtilis		2.9 µg/mL		
nAu@nZnO	*Ruditapes decussatus*	Levels of H_2O_2, MDA, intracellular iron and calcium as well as the activities of SOD and CAT	n.d.	nAu@nZnO hybrid NMs induced biochemical and histological alterations within either the digestive gland or gill tissues at high concentration	Sellami et al., 2017

nAg@MWCNT	*Methylobacterium* spp.	Bacterial growth inhibition	30 µg/mL	30 µg/mL of synthesized Ag@MWCNTs yielded an efficient level of antibacterial activity against *Methylobacterium* spp. and *Sphingomonas* spp.	Seo et al., 2014
	Sphingomonas spp.				
nAu@nAg	*Escherichia coli*	Bacterial growth inhibition	10 µg/mL	Compared with individual nAg and the simple mixture of nAu and nAg, bimetallic nAu@nAg with remarkable stability and a long-term antibacterial efficiency while possessed synergistically enhanced antibacterial activity against both gram-negative and gram-positive bacteria, even at a lower silver concentration	Yang et al., 2017
	Staphylococcus aureus		15 µg/mL		
nAg@GO	*Escherichia coli*	Bacterial growth inhibition	3.2 µg/mL	After conjugating to GO sheets, the antibacterial activities of nAg against *E. coli* and *B. subtilis* were significantly enhanced	Zhu et al., 2013
	Bacillus subtilis		6.4 µg/mL		

[a] N.d. = not determined, Chit – chitosan, CNCs – cellulose nanocrystals, CNF – carbon nanofiber, GO – graphene oxide, IO – iron oxide, MWCNT – multiwall carbon nanotube, PSF – polysulfone, rGO – reduced graphene oxide, SWCNT – single walled carbon nanotube.

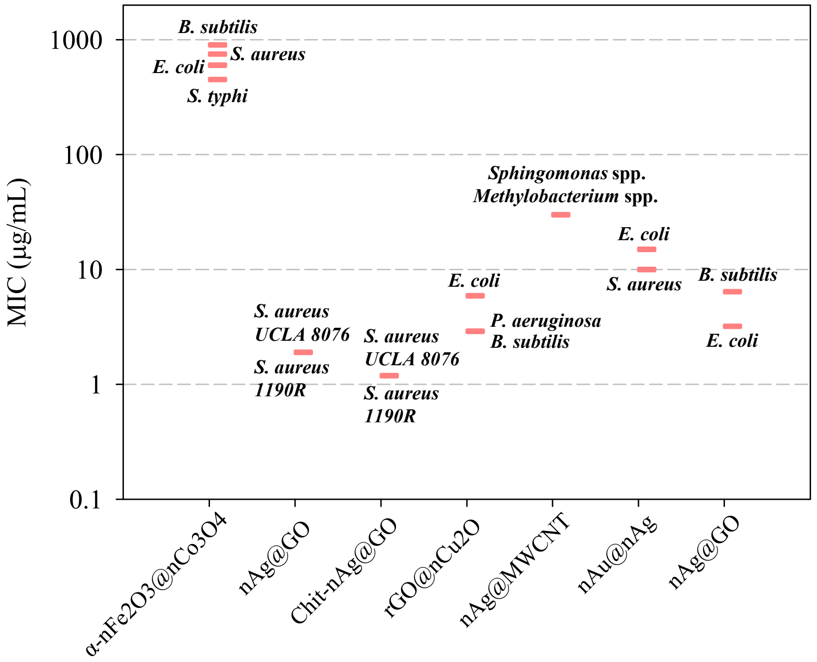

Figure S2. Minimum inhibitory concentrations (MICs) for bacteria exposed to multicomponent nanomaterials.

REFERENCES

Azevedo, S.L., Holz, T., Rodrigues, J., Monteiro, T., Costa, F.M., Soares, A.M.V.M., Loureiro, S., 2017. A mixture toxicity approach to predict the toxicity of Ag decorated ZnO nanomaterials. Sci. Total Environ. 579, 337–344.

Bhaisare, M.L., Wu, B.-S., Wu, M.-C., Khan, M.S., Tseng, M.-H., Wu, H.-F., 2016. MALDI MS analysis, disk diffusion and optical density measurements for the antimicrobial effect of zinc oxide nanorods integrated in graphene oxide nanostructures. Biomater. Sci. 4, 183–194.

Bhushan, M., Kumar, Y., Periyasamy, L., Viswanath, A.K., 2018. Antibacterial applications of α-Fe_2O_3/Co_3O_4 nanocomposites and study of their structural, optical, magnetic and cytotoxic characteristics. Appl. Nanosci. 8, 137–153.

Breisch, M., Loza, K., Pappert, K., Rostek, A., Rurainsky, C., Tschulik, K., Heggen, M., Epple, M., Tiller, J.C., Schildhauer, T.A., Köller, M., Sengstock, C., 2020. Enhanced dissolution of silver nanoparticles in a physical mixture with platinum nanoparticles based on the sacrificial anode effect. Nanotechnology 31, 055703.

Chen, J., Sun, L., Cheng, Y., Lu, Z., Shao, K., Li, T., Hu, C., Han, H., 2016. Graphene oxide-silver nanocomposite: novel agricultural antifungal agent against *Fusarium graminearum* for crop disease prevention. ACS Appl. Mater. Interfaces 8, 24057–24070.

Chen, X., Wilke, C.M., Gaillard, J.-F., Gray, K.A., 2020. Combined toxicity of nano-CuO/nano-TiO_2 and $CuSO_4$/nano-TiO_2 on *Escherichia coli* in aquatic environments under dark and light conditions. NanoImpact 19, 100250.

Choi, O., Hu, Z., 2009. Role of reactive oxygen species in determining nitrification inhibition by metallic/oxide nanoparticles. J. Environ. Eng. 135, 1365–1370.

Da Silva, G.H., Clemente, Z., Khan, L.U., Coa, F., Neto, L.L.R., Carvalho, H.W.P., Castro, V.L.,

Martinez, D.S.T., Monteiro, R.T.R., 2018. Toxicity assessment of TiO_2-MWCNT nanohybrid material with enhanced photocatalytic activity on *Danio rerio* (Zebrafish) embryos. Ecotoxicol. Environ. Saf. 165, 136–143.

Dabour, K., Al Naggar, Y., Masry, S., Naiem, E., Giesy, J.P., 2019. Cellular alterations in midgut cells of honey bee workers (*Apis millefera* L.) exposed to sublethal concentrations of CdO or PbO nanoparticles or their binary mixture. Sci. Total Environ. 651, 1356–1367.

Das, S., Thiagarajan, V., Chandrasekaran, N., Ravindran, B., Mukherjee, A., 2022. Nanoplastics enhance the toxic effects of titanium dioxide nanoparticle in freshwater algae *Scenedesmus obliquus*. Comp. Biochem. Phys. C 256, 109305.

de Medeiros, A.M.Z., Khan, L.U., da Silva, G.H., Ospina, C.A., Alves, O.L., de Castro, V.L., Martinez, D.S.T., 2021. Graphene oxide-silver nanoparticle hybrid material: an integrated nanosafety study in zebrafish embryos. Ecotoxicol. Environ. Saf. 209, 111776.

Estrela, F.N., Batista Guimarães, A.T., Silva, F.G., Marinho da Luz, T., Silva, A.M., Pereira, P.S., Malafaia, G., 2021. Effects of polystyrene nanoplastics on *Ctenopharyngodon idella* (grass carp) after individual and combined exposure with zinc oxide nanoparticles. J. Hazard. Mater. 403, 123879.

Fang, R., Gong, J., Cao, W., Chen, Z., Huang, D., Ye, J., Cai, Z., 2022. The combined toxicity and mechanism of multi-walled carbon nanotubes and nano copper oxide toward freshwater algae: *Tetradesmus obliquus*. J. Environ. Sci. 112, 376–387.

Fasil, D.M., Hamdi, H., Al-Barty, A., Zaid, A.A., Parashar, S.K.S., Das, B., 2021. Selenium and zinc oxide multinutrient supplementation enhanced growth performance in zebra fish by modulating oxidative stress and growth-related gene expression. Front. Bioeng. Biotechnol. 9, 721717.

Haghighat, F., Kim, Y., Sourinejad, I., Yu, I.J., Johari, S.A., 2021. Titanium dioxide nanoparticles affect the toxicity of silver nanoparticles in common carp (*Cyprinus carpio*). Chemosphere 262, 127805.

Hassanein, A., Keller, E., Lansing, S., 2021. Effect of metal nanoparticles in anaerobic digestion production and plant uptake from effluent fertilizer. Bioresour. Technol. 321, 124455.

Hernández-Moreno, D., Li, L., Connolly, M., Conde, E., Fernández, M., Schuster, M., Navas, J.M., Fernández-Cruz, M.-L., 2016. Mechanisms underlying the enhancement of toxicity caused by the coincubation of zinc oxide and copper nanoparticles in a fish hepatoma cell line. Environ. Toxicol. Chem. 35, 2562–2570.

Hernández-Moreno, D., Valdehita, A., Conde, E., Rucandio, I., Navas, J.M., Fernández-Cruz, M.L., 2019. Acute toxic effects caused by the co-exposure of nanoparticles of ZnO and Cu in rainbow trout. Sci. Total Environ. 687, 24–33.

Hong, M., Gong, J.-L., Cao, W.-C., Fang, R., Cai, Z., Ye, J., Chen, Z.-P., Tang, W.-W., 2022. The combined toxicity and mechanism of multi-walled carbon nanotubes and nano zinc oxide toward the cabbage. Environ. Sci. Pollut. Res. 29, 3540–3554.

Hua, J., Peijnenburg, W.J.G.M., Vijver, M.G., 2016. TiO_2 nanoparticles reduce the effects of ZnO nanoparticles and Zn ions on zebrafish embryos (*Danio rerio*). NanoImpact 2, 45–53.

Huang, B., Wei, Z.-B., Yang, L.-Y., Pan, K., Miao, A.-J., 2019. Combined toxicity of silver nanoparticles with hematite or plastic nanoparticles toward two freshwater algae. Environ. Sci. Technol. 53, 3871–3879.

Iswarya, V., Bhuvaneshwari, M., Alex, S.A., Iyer, S., Chaudhuri, G., Chandrasekaran, P.T., Bhalerao, G.M., Chakravarty, S., Raichur, A.M., Chandrasekaran, N., Mukherjee, A., 2015.

Combined toxicity of two crystalline phases (anatase and rutile) of Titania nanoparticles towards freshwater microalgae: *Chlorella* sp. Aquat. Toxicol. 161, 154–169.

Iswarya, V., Bhuvaneshwari, M., Chandrasekaran, N., Mukherjee, A., 2016. Individual and binary toxicity of anatase and rutile nanoparticles towards *Ceriodaphnia dubia*. Aquat. Toxicol. 178, 209–221.

Iswarya, V., Bhuvaneshwari, M., Chandrasekaran, N., Mukherjee, A., 2018. Trophic transfer potential of two different crystalline phases of TiO_2 NPs from *Chlorella* sp. to *Ceriodaphnia dubia*. Aquat. Toxicol. 197, 89–97.

Iswarya, V., Palanivel, A., Chandrasekaran, N., Mukherjee, A., 2019. Toxic effect of different types of titanium dioxide nanoparticles on *Ceriodaphnia dubia* in a freshwater system. Environ. Sci. Pollut. Res. 26, 11998–12013.

Jahan, S., Alias, Y.B., Bakar, A.F.B.A., Yusoff, I.B., 2018. Toxicity evaluation of ZnO and TiO_2 nanomaterials in hydroponic red bean (*Vigna angularis*) plant: physiology, biochemistry and kinetic transport. J. Environ. Sci. 72, 140–152.

Jośko, I., Oleszczuk, P., Skwarek, E., 2017. Toxicity of combined mixtures of nanoparticles to plants. J. Hazard. Mater. 331, 200–209.

Jośko, I., Kusiak, M., Xing, B., Oleszczuk, P., 2021. Combined effect of nano-CuO and nano-ZnO in plant-related system: from bioavailability in soil to transcriptional regulation of metal homeostasis in barley. J. Hazard. Mater. 416, 126230.

Jośko, I., Krasucka, P., Skwarek, E., Oleszczuk, P., Sheteiwy, M., 2022. The co-occurrence of Zn- and Cu-based engineered nanoparticles in soils: the metal extractability vs. toxicity to *Folsomia candida*. Chemosphere 287 (Pt 3), 132252.

Jung, E.S., Sivakumar, S., Hong, S.-C., Yi, P.-I., Jang, S.-H., Suh, J.-M., 2020. Influence of relative humidity on germination and metal accumulation in *Vigna radiata* exposed to metal-based nanoparticles. Sustainability 12, 1347.

Kaur, J., Khatri, M., Puri, S., 2019. Toxicological evaluation of metal oxide nanoparticles and mixed exposures at low doses using zebra fish and THP1 cell line. Environ. Toxicol. 34, 375–387.

Kong, I.C., Ko, K.-S., Koh, D.-C., 2021. Comparisons of the effect of different metal oxide nanoparticles on the root and shoot growth under shaking and non-shaking incubation, different plants, and binary mixture conditions. Nanomaterials 11, 1653.

Li, S., Chang, R., Chen, J., Mi, G., Xie, Z., Webster, T.J., 2020. Novel magnetic nanocomposites combining selenium and iron oxide with excellent anti-biofilm properties. J. Mater. Sci. 55, 1012–1022.

Liu, Y., Baas, J., Peijnenburg, W.J.G.M., Vijver, M.G., 2016. Evaluating the combined toxicity of Cu and ZnO nanoparticles: utility of the concept of additivity and a nested experimental design. Environ. Sci. Technol. 50, 5328–5337.

Liu, Y., Wang, S., Wang, Z., Ye, N., Fang, H., Wang, D., 2018. TiO_2, SiO_2 and ZrO_2 nanoparticles synergistically provoke cellular oxidative damage in freshwater microalgae. Nanomaterials 8, 95.

Lopes, S., Pinheiro, C., Soares, A.M.V.M., Loureiro, S., 2016. Joint toxicity prediction of nanoparticles and ionic counterparts: simulating toxicity under a fate scenario. J. Hazard. Mater. 320, 1–9.

Lozovskis, P., Jankauskaitė, V., Guobienė, A., Kareivienė, V., Vitkauskienė, A., 2020. Effect of graphene oxide and silver nanoparticles hybrid composite on *P. aeruginosa* strains with acquired resistance genes. Int. J. Nanomedicine 15, 5147–5163.

Lu, G., Yang, H., Xia, J., Zong, Y., Liu, J., 2017. Toxicity of Cu and Cr Nanoparticles to *Daphnia magna*. Water Air Soil Pollut. 228, 18.

Malatjie, T.S., Botha, T.L., Kuvarega, A.T., Madima, N., de Bruyn, K., Tekere, M., Nkambule, T.T.I., Mamba, B.B., Msagati, T.A.M., 2022. Toxicity evaluation of TiO_2/MWCNT-CNF hybrid nanocomposites with enhanced photocatalytic activity toward freshwater microalgae: *Pseudokirchneriella subcapitata*. Chemosphere 291, 132891.

Mansouri, B., Maleki, A., Johari, S.A., Shahmoradi, B., Mohammadi, E., Shahsavari, S., Davari, B., 2016. Copper bioaccumulation and depuration in common carp (*Cyprinus carpio*) following co-exposure to TiO_2 and CuO Nanoparticles. Arch. Environ. Contam. Toxicol. 71, 541–552.

Mansouri, B., Maleki, A., Johari, S.A., Shahmoradi, B., Mohammadi, E., Davari, B., 2017. Histopathological effects of copper oxide nanoparticles on the gill and intestine of common carp (*Cyprinus carpio*) in the presence of titanium dioxide nanoparticles. Chem. Ecol. 33, 295–308.

Mantecca, P., Moschini, E., Bonfanti, P., Fascio, U., Perelshtein, I., Lipovsky, A., Chirico, G., Bacchetta, R., Del Giacco, L., Colombo, A., Gedanken, A., 2015. Toxicity evaluation of a new Zn-doped CuO nanocomposite with highly effective antibacterial properties. Toxicol. Sci. 146, 16–30.

Marcone, G.P.S., Oliveira, Á.C., Almeida, G., Umbuzeiro, G.A., Jardim, W.F., 2012. Ecotoxicity of TiO_2 to *Daphnia similis* under irradiation. J. Hazard. Mater. 211–212, 436–442.

Marta, B., Potara, M., Iliut, M., Jakab, E., Radu, T., Imre-Lucaci, F., Katona, G., Popescu, O., Astilean, S., 2015. Designing chitosan–silver nanoparticles–graphene oxide nanohybrids with enhanced antibacterial activity against *Staphylococcus aureus*. Colloid. Surface. A 487, 113–120.

Miranda, R.R., Damaso da Silveira, A.L.R., de Jesus, I.P., Grötzner, S.R., Voigt, C.L., Campos, S.X., Garcia, J.R.E., Randi, M.A.F., Ribeiro, C.A.O., Filipak Neto, F., 2016. Effects of realistic concentrations of TiO_2 and ZnO nanoparticles in *Prochilodus lineatus* juvenile fish. Environ. Sci. Pollut. Res. 23, 5179–5188.

Mocanu, A., Rusen, E., Diacon, A., Isopencu, G., Mustățea, G., Şomoghi, R., Dinescu, A., 2019. Antimicrobial properties of polysulfone membranes modified with carbon nanofibers and silver nanoparticles. Mater. Chem. Phys. 223, 39–45.

Mohammed, M.K.A., Ahmed, D.S., Mohammad, M.R., 2019. Studying antimicrobial activity of carbon nanotubes decorated with metal-doped ZnO hybrid materials. Mater. Res. Express 6, 055404.

Muccifora, S., Castillo-Michel, H., Barbieri, F., Bellani, L., Ruffini Castiglione, M., Spanò, C., Pradas del Real, A.E., Giorgetti, L., Tassi, E.L., 2021. Synchrotron radiation spectroscopy and transmission electron microscopy techniques to evaluate TiO_2 NPs incorporation, speciation, and impact on root cells ultrastructure of *Pisum sativum* L. plants. Nanomaterials 11, 921.

Naeem, H., Ajmal, M., Qureshi, R.B., Muntha, S.T., Farooq, M., Siddiq, M., 2019. Facile synthesis of graphene oxide–silver nanocomposite for decontamination of water from multiple pollutants by adsorption, catalysis and antibacterial activity. J. Environ. Manage. 230, 199–211.

Natarajan, L., Jenifer, M.A., Chandrasekaran, N., Suraishkumar, G.K., Mukherjee, A., 2022. Polystyrene nanoplastics diminish the toxic effects of Nano-TiO_2 in marine algae *Chlorella* sp. Environ. Res. 204, 112400.

Ogunsuyi, O.I., Fadoju, O.M., Akanni, O.O., Alabi, O.A., Alimba, C.G., Cambier, S., Eswara, S., Gutleb, A.C., Adaramoye, O.A., Bakare, A.A., 2019. Genetic and systemic toxicity induced by silver and copper oxide nanoparticles, and their mixture in *Clarias gariepinus* (Burchell, 1822). Environ. Sci. Pollut. Res. 26, 27470–27481.

Pikula, K., Johari, S.A., Santos-Oliveira, R., Golokhvast, K., 2022. Individual and binary mixture toxicity of five nanoparticles in marine microalga *Heterosigma akashiwo*. Int. J. Mol. Sci. 23, 990.

Sayadi, M.H., Pavlaki, M.D., Martins, R., Mansouri, B., Tyler, C.R., Kharkan, J., Shekari, H., 2021. Bioaccumulation and toxicokinetics of zinc oxide nanoparticles (ZnO NPs) co-exposed with graphene nanosheets (GNs) in the blackfish (*Capoeta fusca*). Chemosphere 269, 128689.

Sayadi, M.H., Pavlaki, M.D., Loureiro, S., Martins, R., Tyler, C.R., Mansouri, B., Kharkan, J., Shekari, H., 2022. Co-exposure of zinc oxide nanoparticles and multi-layer graphenes in blackfish (*Capoeta fusca*): evaluation of lethal, behavioural, and histopathological effects. Ecotoxicology 31, 425–439.

Selim, M.S., Samak, N.A., Hao, Z., Xing, J., 2020. Facile design of reduced graphene oxide decorated with Cu_2O nanocube composite as antibiofilm active material. Mater. Chem. Phys. 239, 122300.

Sellami, B., Mezni, A., Khazri, A., Bouzidi, I., Saidani, W., Sheehan, D., Beyrem, H., 2017. Toxicity assessment of ZnO-decorated Au nanoparticles in the Mediterranean clam *Ruditapes decussatus*. Aquat. Toxicol. 188, 10–19.

Seo, Y., Hwang, J., Kim, J., Jeong, Y., Hwang, M.P, Choi, J., 2014. Antibacterial activity and cytotoxicity of multi-walled carbon nanotubes decorated with silver nanoparticles. Int. J. Nanomedicine 9, 4621–4629.

Singh, D., Kumar, A., 2016. Impact of irrigation using water containing CuO and ZnO nanoparticles on *Spinach oleracea* grown in soil media. Bull. Environ. Contam. Toxicol. 97, 548–553.

Singh, D., Kumar, A., 2018. Investigating long-term effect of nanoparticles on growth of *Raphanus sativus* plants: a trans-generational study. Ecotoxicology 27, 23–31.

Singh, D., Kumar, A., 2019. Assessment of toxic interaction of nano zinc oxide and nano copper oxide on germination of *Raphanus sativus* seeds. Environ. Monit. Assess. 191, 703.

Singh, D., Kumar, A., 2020a. Quantification of metal uptake in *Spinacia oleracea* irrigated with water containing a mixture of CuO and ZnO nanoparticles. Chemosphere 243, 125239.

Singh, D., Kumar, A., 2020b. Binary mixture of nanoparticles in sewage sludge: impact on spinach growth. Chemosphere 254, 126794.

Skiba, E., Pietrzak, M., Glińska, S., Wolf, W.M., 2021. The combined effect of ZnO and CeO_2 nanoparticles on *Pisum sativum* L.: a photosynthesis and nutrients uptake study. Cells 10, 3105.

Srivastava, S., Kumar, A., 2017. Comparative cytotoxicity of nanoparticles and ions to *Escherichia coli* in binary mixtures. J. Environ. Sci. 55, 11–19.

Subpiramaniyam, S., Hong, S.-C., Yi, P.-I., Jang, S.-H., Suh, J.-M., Jung, E.-S., Park, J.-S., Cho, L.-H., 2021. Influence of sawdust addition on the toxic effects of cadmium and copper oxide nanoparticles on *Vigna radiata* seeds. Environ. Pollut. 289, 117311.

Tong, T., Wilke, C.M., Wu, J., Binh, C.T.T., Kelly, J.J., Gaillard, J.-F., Gray, K.A., 2015. Combined

toxicity of nano-ZnO and nano-TiO_2 : from single- to multinanomaterial systems. Environ. Sci. Technol. 49, 8113–8123.

Wang, Z., Jin, S., Zhang, F., Wang, D., 2020. Combined toxicity of TiO_2 nanospherical particles and TiO_2 nanotubes to two microalgae with different morphology. Nanomaterials 10, 2559.

Wang, Z., Zhang, F., Vijver, M.G., Peijnenburg, W.J.G.M., 2021. Graphene nanoplatelets and reduced graphene oxide elevate the microalgal cytotoxicity of nano-zirconium oxide. Chemosphere 276, 130015.

Wilke, C.M., Tong, T., Gaillard, J.-F., Gray, K.A., 2016. Attenuation of microbial stress due to nano-Ag and nano-TiO_2 interactions under dark conditions. Environ. Sci. Technol. 50, 11302–11310.

Wilke, C.M., Wunderlich, B., Gaillard, J.-F., Gray, K.A., 2018. Synergistic bacterial stress results from exposure to nano-Ag and nano-TiO_2 mixtures under light in environmental media. Environ. Sci. Technol. 52, 3185–3194.

Xia, J., Zhao, H.Z., Lu, G.H., 2013. Effects of selected metal oxide nanoparticles on multiple biomarkers in *Carassius auratus*. Biomed. Environ. Sci. 26, 742–749.

Yang, L., Yan, W., Wang, H., Zhuang, H., Zhang, J., 2017. Shell thickness-dependent antibacterial activity and biocompatibility of gold@silver core–shell nanoparticles. RSC Adv. 7, 11355–11361.

Yang, Q., Zhang, L., Ben, A., Wu, N., Yi, Y., Jiang, L., Huang, H., Yu, Y., 2018. Effects of dispersible MoS_2 nanosheets and nano-silver coexistence on the metabolome of yeast. Chemosphere 198, 216–225.

Ye, N., Wang, Z., Fang, H., Wang, S., Zhang, F., 2017. Combined ecotoxicity of binary zinc oxide and copper oxide nanoparticles to *Scenedesmus obliquus*. J. Environ. Sci. Heal. A 52, 555–

560.

Ye, N., Wang, Z., Wang, S., Peijnenburg, W.J.G.M., 2018. Toxicity of mixtures of zinc oxide and graphene oxide nanoparticles to aquatic organisms of different trophic level: particles outperform dissolved ions. Nanotoxicology 12, 423–438.

Yin, J., Huang, G., An, C., Feng, R., 2022. Nanocellulose enhances the dispersion and toxicity of ZnO NPs to green algae *Eremosphaera viridis*. Environ. Sci.: Nano 9, 393–405.

Yu, Q., Wang, Z., Wang, G., Peijnenburg, W.J.G.M., Vijver, M.G., 2022. Effects of natural organic matter on the joint toxicity and accumulation of Cu nanoparticles and ZnO nanoparticles in *Daphnia magna*. Environ. Pollut. 292, 118413.

Yu, R., Wu, J., Liu, M., Zhu, G., Chen, L., Chang, Y., Lu, H., 2016a. Toxicity of binary mixtures of metal oxide nanoparticles to *Nitrosomonas europaea*. Chemosphere 153, 187–197.

Yu, R., Wu, J., Liu, M., Chen, L., Zhu, G., Lu, H., 2016b. Physiological and transcriptional responses of *Nitrosomonas europaea* to TiO_2 and ZnO nanoparticles and their mixtures. Environ. Sci. Pollut. Res. 23, 13023–13034.

Zain, N.M., Stapley, A.G.F., Shama, G., 2014. Green synthesis of silver and copper nanoparticles using ascorbic acid and chitosan for antimicrobial applications. Carbohyd. Polym. 112, 195–202.

Zhang, H., Shi, J., Su, Y., Li, W., Wilkinson, K.J., Xie, B., 2020. Acute toxicity evaluation of nanoparticles mixtures using luminescent bacteria. Environ. Monit. Assess. 192, 484.

Zhang, Y., Li, X., Liang, J., Luo, Y., Tang, N., Ye, S., Zhu, Z., Xing, W., Guo, J., Zhang, H., 2022. *Microcystis aeruginosa*'s exposure to an antagonism of nanoplastics and MWCNTs: the disorders in cellular and metabolic processes. Chemosphere 288, 132516.

Zhao, H-Z., Lu, G-H., Xia, J., Jin, S-G., 2012. Toxicity of nanoscale CuO and ZnO to *Daphnia magna*. Chem. Res. Chin. Univ. 28, 209–213.

Zhao, J., Dai, Y., Wang, Z., Ren, W., Wei, Y., Cao, X., Xing, B., 2018. Toxicity of GO to freshwater algae in the presence of Al_2O_3 particles with different morphologies: importance of heteroaggregation. Environ. Sci. Technol. 52, 13448–13456.

Zhu, X., Tan, L., Zhao, T., Huang, W., Guo, X., Wang, J., Wang, J., 2022. Alone and combined toxicity of ZnO nanoparticles and graphene quantum dots on microalgae *Gymnodinium*. Environ. Sci. Pollut. Res. 29, 47310–47322.

Zhu, Z., Su, M., Ma, L., Ma, L., Liu, D., Wang, Z., 2013. Preparation of graphene oxide–silver nanoparticle nanohybrids with highly antibacterial capability. Talanta 117, 449–455.
